# Supplementary material for: The Functional Characterization of DzCYP72A12-4 Related to Diosgenin Biosynthesis and Drought Adaptability in Dioscorea zingiberensis
Source: Int J Mol Sci. 2023 May 8;24(9):8430. doi: 10.3390/ijms24098430 (PMC10179397; doi:10.3390/ijms24098430)
Supplement: Supplementary file 1 [file ijms-24-08430-s001.zip › Table S2.pdf]

Table S2. Protein sequences used to construct the phylogenetic tree.

>KAJ0960429.1

MEEMLELLWAWIWPLVWLSLSLVMVMKVLEVFYWRPKRIEKHFSRQGIKGPSYRFFIGNVKEMVSLIFEASSKPMMPCNS
HNILPRVLSFYHHWKKIYGSTFVLWFGPIARLTVADPDLIREIFVSRSEYFERYEAHPLVRQLEGDGLISLRGEKWAHHR
KVITPTFHMDNLKLLMPLIGKSMVEMVEKWSSVSSSGDFEIDVYDWFHSLTEDAISQTVFGRSYEDGKAVFQLQAQQMLF
AAEAFYKVLIPGYRLISRRKDDHDDEEKADDDGRAKDLLGLMISESRKRFPLRLSGPSSTIISVQDIMEECKTFFFAGKQ
TTSSLLTWATVLLAMHSDWQELARQEVLSVCGSSDIPTRDHLSKLKTLGMVLYETLRLYPPAVATIRSARADVDLGGYHI
PRGTELLIPIIAVHHDTSLWGSDAAEFNPARFAGGVALAAKHPAAFIPFGLGARTCIGQNLALLEAKLTVAILLQRFSFR
LAPSYVHAPTVLMLLYPRYGSPVIFRRLSSNQHNHPST

>KAJ0961056.1

MDDYLLQLQSIAALFLFVFLFNLWIQKTFRSKNKEPPQASGGFPVIGHLLLLRGSGRPLYQVLGDMADKHGPAFILRLGS
RRTLVVSNCEVAKECFTINDKALSSRPANATARHLGYNLAMVGFAPYGSYWRSLRKISTTELLSNARLDMLKHVISGEID
TCLNELSTHCLCNDNNSPVKVDMMKWFGDLNFNIVLQMVTGKRFFGSGGGSDEAWRFRNAVTKFFHHLSVSVPSNMFPWL
EWMDLGGHVKAMKAVAKEMDSVMVSLLEEHREMRASGLAAAGDRDFMDVMLSHIESDQFKELHDKDTVIKATSLAMLLGG
TDTTTTSLTRVLMNLLKNEEVMKKVQTELDEQVGKDRVVNESDMKKLVYIQAVIKESFRLTPSAELLVPRETMEDCVVAG
FQVPAGTQVIVNAWKIQRDPKVWPDPSEFRPERFLESHAAGIDVKGQNYELIPFGTGRRSCPGVSMALHVMNLTLARLIQ
GFKLSPVGDLTPATAAPVGGYSVQVYGFGGYGVQLVKKSDARSRVDRESDNHQLMLLDEIDVVSSVGTVLSCRCGPGEWM
PMVHHAQNMWYR

>KAJ0961057.1

MEYSLLDLLSILGVFAFAFLCHKWIKNLTRTNTNVPPQASGALPVIGHLHLLRGPKPVFKLLADMADKYGPAFTIRLGSV
RTLILSNWETTKDCFTTNDIVVAARPVNAATEYLGHNYAMFGFAPYGAYWRAVRKITLTDLLSPARLNMLKHVPAAEVDT
CMKELFQLCNSCDQNPNHVAKVDMKQWFGHLNYNIVVQMVAGKRYFGSGSASEEAWRFRDAATQFFHLLLSFVPSDMFPF
LAWMDIGGYVKAMKKASKEMDDVMDGLVEEHRKRRACGEVNEHPDFIDLMLSALEGEKLEGFDWKTVIKTTSLTLILGGT
DTTSTSLTKVLANLLNHREVLKKVQEEIDEQVGRDRVVNESNTKKLVYLQAVIKESFRMTPPDPFLIRRATQKDCILAGY
HVPKGTQVIVNAWKVHNDPNVWPEPSEFRPERFLSSQKGIDVKGQHYELIPFGTGRRVCPGVTMALQVMNLTLARLIQGF
ELRTPGDVPVEIEGGLVSLASDSDPFMVELTPRLSPELY

>KAJ0961255.1(DzCYP72A23)

MESVMGVIWAVAAVVVVAAAWRTLDWIWWTPRRLDRELRRQGLRGNQYRVLHGDLKENVRLSKEAKSRPLPLHCHDIAPR
VLPLFHNAIKDHGKISITWLGPYPRVTLTEPELVKEVLSNKFGHFVKPTTNPLAKFLIQGLASYEGEKWAKHRRIINPAF
HLEKLKLMLPAFSTSCGELIRRWEKMIPNEGSQELNVFPELQDLTKDVISRTAFGSSYEDGRRIFELLTEQIQLLIPAFQ
TVYIPGYRFLPTPMNKRSSQVYNEMKRILIGMIEKREKAIRMGESSKNDLLGLLLDSNIKESEVHGKSQNKGMSTEDVVE
ECKLFYFAGQETTSLLLTWTMILLSMYPNWQAKAREEVLQVFGKSAPDMEGLSHLKIVTMILYEVLRLYPPAVFLTRKTY
KAMELGGITYPPGVILSLPLLFIHHDPVFWGEDAKEFNPERFSEGISKASKVPGAFFPFGGGPRICIGQNFALIEAKIGI
CMILQHFSFVLSPSYIHAPHNVITLQPQHGAQLMLQKV

>KAJ0961256.1(DzCYP72A24)

MELVMGVIWTVTAAVVVVWAWRTLDWVWWTPRRLDRDLRRQGLRGNQYRLLHGDLKENARLSKEATSRPLPLHCHEIAPR
VAPLIHNAIKDHGKISITWLGPYPRVSLMDPDLVKEVLSNKFGHFVKPRITPIAKFLVQGLVAYEGEKWAKHRRIINPAF
HLEKLKLMLPAFSTSSGELIRRWEKMIPDEGSQELNVSPEIQNLTGDVISRTAFGSSYEEGRRIFELLTEQIHLTIPALQ
TVYIPGYRFLPTPMNNRRSQVYNEMKRILKGMIEKREKAIRMGESSKNDLLGLLLDSNMKEGEEQEKSQNKVMSTEDVIE
ECKLFYFAGQETTSVLLTWTMILLSMYPNWQAKAREEVLQVFGKSTPDMDGLSRLKIVTMILYEVLRLYPPVTFLTRKTY
KAMELGGINYPPGVLLQLPLLFIHHDPEFWGEDAKEFNPERFSEGILKASKVPGAFFPFGGGPRICIGQSFALIEAKIGI
SMILQHFSFELSPSYIHAPHTPLTLQPQHGAQLMLQKL

>KAJ0961259.1(DzCYP72A25)

MEYSVAAGVKEMIWGVATALLVVWVWRTLEWLWWKPRRLERELKRQGLRGGKYRLFHGDLKDNARLMKDALSRPLPPYSH

DVAQRVIPLVHQAIKIHGKMSITWLGPYPRVSLMDPELIREVLSNKFGHFVKPNFSPLVKLLAQGLASHEGEKWAKHRRI
INPAFHFEKLKCMLPAFSTCCDELVKRWQNKMNVEGSIELNMWPELQNLTGDVISRTAFGSCYEEGRRIFQLQTEQAELI
IPTAQTVYYVPGFRFLPTPKNNRRKAIDREIKTILRSMIEKREKAIRMGGETSCKDDLLGLLLESNMKETEQQGRSKNKGL
TTEDVIEECKLFYLAGQETTSSLLTWTMVLLSMYPNWQAKAREEVLQVFGKNKPDMDGVGRLKIVTMILYEVLRLYPPAI
FLTRRTYKTMELGGITYPPGVLLLLPIILVHHDLDFWGEDAKDFKPDRFAEGISKASKVPGAFFPFGGGPRICIGQSFAL
IEAKLGLSMILQNFSFELSPSYIHAPYTLVTLQPQHGAPIKLHKL
>KAJ0962283.1
MEEELGWAWPAVQGAWRYVMAFVAVGMSLVMVMKVLELLWWRPRRLEEHFARQGIRGPPYKFFLGNVKEMVGYMLEASSK
PMMPHNSHNILPRVLSFYHHWKKIYGSTFLLWFGPTARLTVADPDLIREIFVSRADSFERYESHPLVRQLEGDGLVSLRG
AKWAHHRKVLTPTFHMDNLKLLIPYIGKTVVDMVDKWLAIPTESGEVEIDVSEWFQTVTEDAITRTAFGRSYDDGKAVFQ
LQGRQMVFAAEAFRKRNTSSWRLDKEIKKDLVGLIERRKARRRRSSDEVEEEKTNDEDVCPKDLLGQMINASTKTPSSDA
ITDHDIVEECKTFFFAGKQTTSNLLTWTTVLLAMHPEWQDLARQEVLRVCGSRDIIPSRDHLAKLKTLGMILNETLRLYP
PAVATIRRAKADVELGGCKIPRGTELLIPIIGVHHDTKLWGPDAARFNPARFGEGARAAKHPTAFIPFGLGARMCIGKNL
ALLEAKLTVAIILQRFSFRLAPSYVHAPTVLMLLYPQYGAPVIFRPLSSPPSITDDSSFQ
>KAJ0962625.1(DzCYP72A16)
MVVVVVFVSLVVIVGLVARVSWWIWWKPKMMEKEMRRQGINGTKYRTFMGDLKDEGEAYKEAWSKPMDLNHNIVPRVLPH
AHLMLQTYGKTSFKWMGTTPAVTIWEPELLREVLMNKCGHFVQPQVNPLIKLLANGINSLEGEQWAQRRKMIKPAFHIDK
LKEMVPTFITSCSELIERWKKLLTDETSCELDVWPEFQNLTIDAISRILFGNSFEEGKRIFELQKEQIVLAIDAASSIYV
PGFRFLPTTKNKRRMFIDNEIKRILQDIINNKLESMKMGGNANSDILSLMLQYGNINIAEKEKNTNNSKITIDNVIEECK
GFYFAGQDTTSTLLTWTLILLSMHPTWQQKAREEVLRTCGKNTPNFESINHLKIVNMILHEVLRLYPPVITQIRHTEKKT
KLGDITLPAGVHVLIPTLQVHHDQEFWGEDAEEFNPERFSEGVSKASKGQNAFLPFGWGPRMCLGQAFAMIETKLGLAMV
LQHFSFELSPSYAHAPSIVATLQPQLGAHLILHQL
>KAJ0962630.1(DzCYP72A17)
MLEMQLRRQGLPGNKYRLMMGDMKDEKKSFKEAWSRPMELTHRIAARVIPYDHQMAQTHGKISFKWNGTTPRVNIWNPEM
SREILLNKSGHIIKPQLNPLIRLLTMGVSTLEGEEWAQRRKLINPAFHMEKLKEMVPVFRISCIDLVKRWENLVSAEGSC
ELDVWPEFQSLTGDVISRTAFGSSFEEGKQIFELQKEQAVLVIEASRSIYLPGFRFLPTAKNKRRMFIDSEIKRMLRDII
HKKLDSMKIGENADDDLLSLLLQSDTMNVVAEDKNKKNNGITIDDVIEECKLFYFAGQEGTSILLTWTLILLSMYPSWQK
KAREEVLNNCGKNTPEFENISHLKIVNMILHEVLRLYPPGVTLIRYINKKVKVGNITLPAGAEVLIPILQVHHDPEIWGE
DAEEFKPERFSEGVSNASKGQQAFFPFGWGPRICSGQTFAMIEAKLALAMVLQNFSFELSPSYTHAPYNVITLQPQYGAH
LILHQL
>KAJ0962634.1
MNVATEDKNKKNNGITIDDVIEECKLFYFAGQETTSILLTWTLILLSMYPTWQKKAREEVLNTCGKNTPEFENISHLKIV
NMILHEVLRLYPPVITLFRHINKNVKLGDITLPAGAEVLIPILQVHHDPEIWGEDAEEFKPERFSEGVSNASKGQNAFFP
FGWGPRILIGVLGRVSYLVWWKPKDAGDAAEKTGVARNKYRLMMGDMKDEKKSFKEAWSRPMELTHRIAARVIPYDHQMA
QTHGKISFKWNGTTPRVNIWNPEMSREILLNKSGHIIKPQLNPLIRLLTMGVSTLEGEEWAQRRKLINPAFHMEKLKEMV
PAFRISCIDLVKRWENLVSAEGSCELDVWPEFQSLTGDVISRTAFGSSFEEGKQIFEFQKEQAVLVIEAARSIYLPGFRF
LPTAKNKRRMFIDSEIKRMLRDIIHKKIDSMKIGENTDDDLLSLFICNPIL
>KAJ0962638.1(DzCYP72A20)
MGVLWRVLYLVWWKPKMLEMQLKRQGLPGNKYRLMMGDMKDEKKSFKEAWSRPMELTHRIAARVIPYDHQIMAQTHGKIS
FKWKGTTPRVNIWNPEMLKEILLNKSGHIIKPQVNPLIKLLTMGVSTLEGEEWAQRRKLINPAFHMEKLKEMVPAFRISC
IDLVKRWENLVSAEGSCELDVWPEFQSLTGDVISRTAFGSSFEEGKQIFELQKEQAVLVIEAARSIYLPGFRFLPTAKNK
RRMFIDSEIKRMLRDIIHKKIDSMKIGENTDDDLLSLLLQSDTMNVATEDKNKKNNGITIDDVIEECKLFYFAGQETTSI
LLTWTLILLSMYPTWQKKAREEVLNTCGKNTPEFENISHLKIVNMILHEVLRLYPPVITLFRHINKNVKLGDITLPAGAE
VLIPILQVHHDPEIWGRMLKSSKPERFSEGVSNASKGQNAFFPFGWGPRICIGQTFAMIEAKLALAMVLQHFSFDLSPSY

THAPYTVITLQPQYGAHLILHQL

>KAJ0962639.1(DzCYP72A21)

MLEMQLRRQGLPGNKYRLMMGDMKDEKKSFKEAWSRPMELTHRIAARVIPYDHQMAQTHGKISFKWNGTTPRVNIWNPEM
SREILLNKSGHIIKPQLNPLIRLLTMGVSTLEGEEWAQRRKLINPAFHMEKLKEMVPVFRISCIDLVKRWENLVVSAEGS
CELDVWPEFQSLTGDVISRTAFGSSFEEGKQIFELQKEQAVLVIEAARSIYLPGFRFLPTAKNKRRMFIDSEIKRMLRDI
IHKKIDSMKIGENTDDDLLSLLLQSDTMNVATEDKNKKNNGITIDDVIEECKLFYFAGQETTSILLTWTLILLSMYPTWQ
KKAREEVLNTFGKNTPEFENISHLKIVNMILHEVLRLYPPVITLFRHINKNVKLGDITLPAGAEVLIPILQVHHDPEIWG
EDAEEFKPERFSEGVSNASKGQNAFFPFGWGPRICIGQTFAMIEAKLALAMVLQHFSFDLSPSYTHAPYTVITLQPQYGA
HLILHQL

>KAJ0962640.1(DzCYP72A22)

MLEMQLRRQGLPGNKYRLMMGDMKDEKKSFKEAWSRPMELTHRIAARVIPYDHQMAQTHGKISFKWNGTTPRVNIWNPEM
SREILLNKSGHIIKPQLNPLIRLLTMGVSTLEGEEWAQRRKLINPAFHMEKLKEMVPVFRISCIDLVKRWENLVSAEGSC
ELDVWPEFQSLTGDVISRTAFGSSFEEGKQIFELQKEQAVLVIEASRSIYLPGFRFLPTAKNKRRMFIDSEIKRMLRDII
HKKLDSMKIGENADDDLLSLLLQSNTMNVAAEDKNKKNNGITIDDVIEECKLFYFAGQEGTSILLTWTLILLSMYPSWQK
KAREEVLNNCRKNTPEFENISHLKIVNMILHEVLRLYPPGVTLIRYINKKVKVGNITLPAGAEVLIPILQVHHDPEIWGE
DAEEFKPERFSEGVSNASKGQQAFFPFGWGPRICSGQTFAMIEAKLALAMVLQNFSFELSPSYTHAPYNVMTLQPQYGAH
LILHQL

>KAJ0962805.1

MDLTTLHFILIFVFPISIIFLFSNISSKQQQSQPSNGLLRPYPIVGNLPQLLRHRHRFLDWMTELLSASPTNTISFHVLS
NVRGIITANPSNVEHILKTNFDNYPKGPRFIHHLQDFLGRGIVNVDGHLWRVQRKTVSFEFNTRSLRNFVVVNVQHEILS
RLLPLLRKSSRTGAAIDLQDVLERFAFDNICKFAFNEDPVCLSDHDDHVSENNLLSRFAQAFKSAAELSAGRFTYAVPWF
WIITRLLNIGTERQLRESIATVHAFATQIIRSRKKEKNLHDRADDLLSRFISNEDNTDDELLRDIIISFTFAGREGTSSA
LTWFFWLLSRHPEVEHKILQEISAVRAARRQGGGDNSTETFEFEELREMQYLHAAISEAMRLYPPLFVNSTMCQSDDVLP
DGTLIKKGMFVVFSAYCMGRMESIWGKDCLEFKPERWLDGEGAFRNESPYRYPVFHAGPRMCLGKEMAYIQMKSIAAFVL
ERFMVDVLHKDKEPERVPSLTLKMKHGLFVGIRDRN

>KAJ0964198.1(DzCYP94N8)

MEFTWLLLLVLFITTTIFLLLHLNPTPTPNFTPLKPYPILQNLPHLVKNSHRLLFFVTELVSSSPSSTSTLIPFVFTSNP
SNVEHMLRSNFPNYIKGSSVISTLHDFLGDGIFNSNGPLWRLQRKTASFEFNTKSLRSFIFHHVRHESLHALLPILSNTS
RASLPVLIDLQDLLERFAFDNVCSLVFGHDPRCLHDSADGLRFFHAFQEASHLSIERMNHAFDLLWKVNKWLNVGSERRL
NHSLLIVREYASRFVSLRKTQAGDDLLSRFAADETISDDLLVDILICFVLAGRDTTPAALSWFFWLLSSRPDVSRNILVE
IQSIRARSGDRDGDRFFSLEELREMNYLHAALSEAMRLYPPVPLLPRCAAEDDVLPDGTVVKKGWTLMYNAYAMGRMESI
WGKDCMEMRPERWLEDGVFQPTSPFRYPVFLGGPRMCLGKEMAYIQMKAVAACILEKFDIDVVGASGEPQLSVTMTMKGG
LPVRIKERNPCTKAV

>KAJ0965356.1

MELTSPILVSSIILISTILFLLLLQRRRTTRKQTIDGAPTLTFKDLVKNGHRFLDWTTEMLLSSPTNTITLPSTVATSNP
SNIEHILKYNFSNYPKGHNITDTLHDLLGDGIFNTDGDHWKLQRKIASLQFNTKSIRSFVTNAVQIEVTTRLLPLLAAAA
VSGEPIDLQDTLERFSFDNVCKVAFDVDPARLAGDAMDGGRFARAFDTAAEISTNRFRQPRFFWLLRRKLNLGEERRLKE
AVRTVNEFAMKVVHDKKLKGKAGDDDLLSKFIEDDSEHSDEFLRDIIISFVLAGRDTTSSALTWFFWLVSSRPEIRRAIR
DEVSAVRAKHGSEPGQELKLEELREMEYLHAALSETLRLYPPVSLEPRACLADDKLPDGTEVKKGWSVMYNSYAMGRMKS
IWGEDCMEFRPERWLVNGEFQAKSPFKFPIFHAGPRTCLGKEMAYIQMKAVAASVLERWLVNGEFQAKSPFKFPIFHAGP
RTCLGKEMAYIQMKAVAASVLERFELEMAPGEEKERAHGFTIVLRMNGGLPIVVKNRD

>KAJ0966589.1

MASTIAFSPSLHLPRPPNAHSRLVSTPRSRSIRCASSNGRGPEEPQGVKDVERILQEKRRAELASRIASGEFTAPQSRLK
SALGRLGPVGKFLDEMLERLAGGDDAKRLKIPQATGSIGAVGGQAFFIPLYELFLTYGGIFRLTFGPKSFLIVSDPAIAK

HILRDNALGYSKGILAEILEFVMGKGLIPANYEVWKVRRKAIVPALHQKYVAKMIGLFGEASYSLCEKLDKAASDGEDVE
MESLFSRLTLDIIGKAVFNYDFDSLTHDNGIIEAVYTVLREAEMRSTSPIPTWEIPIWKDISPRQKKANQALKLVNDTLD
DLIAICKRMVEQEELQFHEEYVNEQDPSILHFLLASGDDVSSKQLRDDLMTLLIAGHETSAAVLTWTFYLLSKEPSIMSK
LQDEVDSVLGDRLPTLEDMKKLKYTTRVINESLRLYPQPPVLIRRSLDDDILGGFPIKSGEDIFISVWNLHRSPKHWVDA
DCFNPERWPLDGPNPNEINQNFSYLPFGGGQRKCIGDMFATFETVVATAMLVRRFNFQMALGAPPVEMTTGATIHTTEGL
LMTVTRRTRPPIIPKLETFTQIVNVDEPVALGEDQQGEVSAASI
>KAJ0966999.1
MELTLLLLLLAFISLLLLLKRKSSPTTANFTPLRRYPFIGNIPHLLNNRSRMLAWASELIPLSPTATSTVAPFVFTANPS
NVEHIIRSNFDNYRKGGFITAAMHDFLGQGILNSNGDLWHLQRKTASFQLNTKSIRSFIMDIVRREAVHSLLPLLHNATT
TGEVLDLQDLLERFAFDNVCNFAFGYDPKSLDGTSEEGLKFFHAFDDATQIVVDRIESIVPWQVKKLLNVGSEKRFREAQ
AIVQEMVSRFVRSTSKQDDLLSRFAEDPNHSEELLRDIGINFMVAGRDTTPSALTWFFWILSSRPHIVEQILNEIKSIKA
RTNINKNSELFGIEELRELHYLHAALSESMRLYPPVPLLPRLANEDDVLPDGTRVRGGWLVMYSSYVMGRTASIWGEDCM
EVKPERWLVDGVYQPTNPFKYTVFHGGPRACLGREMAYIQMKAVAACILERFEMEVVGKRGEPQISMTMRMKGGLPVRIR
ERVLTSLVQEV
>KAJ0967000.1
MHDFLADGIFNSNGELWRLQRKTASFEFNTKSIRSFIMDIVRREAVHSLLPLLHNATTTGEVLDLQDLLERFAFDNVCNL
AFGYDPKSLDGTSEEGLKFFHAFDDATQIIVDRMVSIVPWKVKKLLKVRSEKRFREAQAIVQEMVSRFVRSTRKQDDLLS
CFAENPTHSEELLRDIGINFMVAGRDTTPSALTWFFWILSSRPHIVEQILREIKSIRARTNNENEFFRIESLREMHYLHA
ALSESMRLYPPVPMLPRMANKDDVLPDGTSVRRGWVVTYSSYAMARLKSIWGEDCMEVKPERWLVDGVYQPTSPFKYPVF
HGGPRVFLGKEMAYIQMKAVAACILERFELEVVEKRGERQISVTMRMKGGLPVRIRERMITTL
>KAJ0967001.1
MELSTLLLLLLLPFVYLLLMRRRSSPLAANFTPLRRYPIIGNIPHLLKNRSRMLDWASELIPLSPTATSTVAPFVFTAN
PSNVEHVIRANFDNYPKGGFITSSMHDFFGDGIFNSNGELWRLQRKTASFEFNTKSIRSFIMDIVRREAVHSLLPLLHNA
TTTGEVLDLQDLLERFAFDNVCNLAFGYDPKSLDGTSEEGLKFFHAFDDATHIVVHRTHSLIPWQIKKLLNVGSEKRFRD
SQAIVQEMVSRFVKSTHKQDDLLSRFAEDPTHSEELLRDISINFMVAGRDTTPSALTWFFWILSSRPHIAEQILREIKSI
RARTNNDNDNEFFRIEELREMHYLHAALSESMRLYPPVPLLPRLANEDDVLPDGTSVRRGWFVMYSSYSMARSKSIWGED
CMEVKPERWLVDGVYQPTSPFKYPVFHGGPRVCLGKEMAYIQMKAVAACVLERFELEVVEKRGEPQLSMTMRMKGGLPVR
IRERIITTL
>KAJ0967143.1
MSFSSSLLPSLLPLFPSSSPKPCHSLHPRSSILVPRAYSGPDKPPPTTTTNGSWVSPDWLTSFARSFSPSDDSSIPIADA
RLDDVSDLLGGALFLPLFKWMKENGPVYRLAAGPRNFVIVSDPAIAKHILRNYNNYAKGLVSEVSQFLFGSGFAVAEGSH
WMVRRKAVAPSLHKKFLSVMVDKVFCRCALRLVEKLEPYTLSGDAVNMEQKFSQLTLDVIGLSLFNYNFDSLTTDSPVID
AVYTALKETEARSTDILPYWQIDFLCKIVPRQIKAERAVSIIRKTVEELITKCKELVEAEGEQVDGEDYVNDADPSILRF
LLASREEVTSVQLRDDLLSMLVAGHETTGSVLTWTLYLLSKDSSALFRAQQEVDSVLQGRLPRYDDVKELKYLTRCIYES
MRLYPHPPVLIRRAQAADVLPGNYKVNAGQDIMISVYNIHHSPQVWDRAEEFVPERFDLEGPAPNEANTDFRFIPFGGGP
RKCVGDQFAMLEAIIALAIFLQHMNFELVADQKIGMTTGATIHTTNGLYMTLRQRKLQNDLAHLEDQDVNLQPSLSKD
>KAJ0967765.1(DzCYP90G6)
MFPLAIIVLLFPTLLLLFIGVALGLRSGANESWKKRGLNIPPGSMGWPLLGETIAFRKLHPCTSLGEYMEDRLQRYGKIY
RSNLFGAPTVVSADAELNRFVLMNDGKLFEPSWPKSVADILGKTSMLVLTGEMHRYMKSLSVNFMGIARLRNHFLGDSER
YILENLATWKEGVPFPAKEEACKITFNLMVKNILSMNPGEPETERLRILYMSFMKGVIAMPLNFPGTAYRKAIQSRATIL
KTIEHLMEDRLEKKKAGTDNIGEADLLGFILEQSNLDAEQFGDLLLGLLFGGHETSSTAITLAIYFLEGCPKAVQELREE
HLNLVRMKKQRGESKALTWEDYKSMDFAQCVVSETLRLGNIIKFVHRKANTDVQFKGYDIPSGWSVIPVFAAAHLDPTVY
DNPQKFDPWRWQTISSSTARIDNYMPFGQGLRNCAGLELAKMEIAVFLHHLVLNFDWELAEPDHPLAYAFPEFEKGLPIK
VRKLSILE

>KAJ0967766.1

MGLMGPLLLTLAALAVTVFLLRRRRQPSSKTSKPLASSGTLSELMKNGHRILDWTTELLSSSQTGTVTTFMGVVTANPSN
VEHILKSHFPNYPKGSHSTTILSDFLGAGIFNSDGEHWRLQRKTASLEFTTKSIRSFVSSNVRLETSSRLLPVLHSFARS
GQIVDLQDLFDCLAFDNVCQVTFGYDPARLDSSGDPDSVAFSRAFDRATALSVRRFSHPFPFTWKLLRFLNAGYERELKA
EVAKVHRFAMQVVRRRKKDGDLGDDLLSRFIAEADYSDEFLRDIIISFVLAGRDTTSATLTWFFWLIASRPEVKARVLDE
IRAAREQERERTGTATSEAVLTLDQVRGMDYLHAALSETLRLYPPVPLQTRACAEDDLLPDGTPVKKGSTVMYSAYAMGR
SESIWGEDWKEFRPERWLENGVFRPASSFRFPVFHAGPRMCLGKDMAYIQMKAVAAAVMERFELEVVDEEKPREPEFTMI
LRMKGGLPVRIREKEF

>KAJ0967767.1(DzCYP90B71)

MAPMELLLIVSPLVLALIIFFSFRGTSKGGDKAEKIPPGTMGWPLIGHTIPFMQPHSSASLGLFVDQNIAKHGRIFRMNL
LGKPTIVSADADFNRFILQSEGRMFENSCPTSIAEIMGRWSMLALAGDVHREMRSIAVNFMSNVKLRTYFLPDVEQQAIK
ILSAWRHGSTFSAQEEGKKFAFNLMVKHLMSMDPGMPETEQLRKEYITFMKGMASIPLNLPGTAYRKALQSRSIILKIMG
QKLDERVEKVKRGCEGLEEDDLLASVAAQSNITRDQILDLILSMLFAGHETSSAAICLAIYFLESSPKALQQLREEHINI
AKMKKEKGETGLTWDDYKQMEFTHCVINETLRLGNIVKFLHRKAIKDVQYKGYDIPCGWEVVPIISSAHLDPSIYDDPQS
YNPWRWQTISTATSKNNNIMSFSGGPRLCPGAELAKMEMAVFLHHLVQKFNWELAEHDYPVSFPFLGFPKHLPIKVHAID
HKASA

>KAJ0974988.1(DzCYP72A15)

MEPSFISMAATLSSLLLLYCALTVVHVVWWRPRMIEKQLKKQGIKGRPYKVLRGDLSDIVKIMKEALSKPMELHHHISPR
ALPFVHSTVEQFGKLSIIWYGKNPRIIIQDVELIKEVLANKNGNFLKPLLNPLQRLLAEGVSLVEGDKWVQHRNILNPAF
HLAKLKGMVPAFCTSCSEMISKWEMLFGPEGSCELDVWIELKALTADVISRTAFGSNYREGQKVFEFQQEQIQLMMEASW
IPYIPGFRFLPTKKNRRRYYLDNEIKAIIRSLIHKKEKSMEVGESGGEDLLSLLLQSNHNVHENAANGLKLEGLTIDEVI
EECKLFYFAGHDTTSSLLTWTLILLSMYPAWQTRAREEVHRICGKNMPDYESIGQFKIVTMILHEVLRLYPPVTGQYRHV
YHETKLGELSLPAGVDLFVPSLLVYHDPEIWGEDCKEFNPQRFSEGVLKATKNQFVYFPFGFGQRTCIGQNFAMIEAKIA
LAMMLQHFSFDLSPSYAHCPYSLITLQPQHGAQLIVHRL

>KAJ0975913.1

MLLSALSKLPSPINGGFLGFHSVVLDSVTQTPLASPLTLTKKRSRFRCQSTGTEETKTKRNLLDNASNLLTNLLSGGNLG
SMPVAEGAVSDLFGRPLFFSLYDWFLEHGSVYKLAFGPKAFVVVSDPIVARHVLRENTFSYDKGVLAEILEPIMGKGLIP
ADLDTWKLRRKVIVPGFHSSFLEAMVSVFTNCSERTMLKFEELIEREKPGEKTIELDLEAEFSSLALDIIGLGVFNYDFG
SVTKESPVIKAVYGTLFEAEHRSTFYIPYWNLPFARWIVPRQRKFHNDLKIINDCLDDLIKNAKKTRQETDVEKLQQRDY
SSLKDASLLRFFVDMRGVDVDDRQLRDDLMTMLIAGHETTAAVLTWAVFLLAQNPSKMRKAQAEIDSVLGQGKTTLECIK
KLEYIRLIIAEALRLYPQPPLLIRRALRPDVLPGGYKGLDVGYEIPAGTDIFISVYNLHRSPYFWDRPNDFEPERFLVPK
KSEGIQGWAGFDPGRSPGAMYPNEIIADFAFLPFGGGPRKCVGDQFALLESTVALAMLLQRFDVELRGSADEVELVTGAT
IHTKKGLWCRLKKRTSKTHSNNPNTQTKGV

>KAJ0977987.1

MELILISLFMIIPLSILIFLLPSNGGNSHTLRPYPVIGNLLQFLRHRHRFLDWMTDLLTASPTNTITFRRLGTSGIVTAN
PANVEHLLKTNFHAFPKGPRSFAHLHDFLGLGIFNVDGDLWRLQRKTASFEFNTRSLRTFVLRNVHHETLTRLLPLLRRI
ATECSAVDIQDLLERFAFDNVCKVAFNEDPACLSEAAENVPRLSTEFAQSFRDAAELSSGRFRYALPWFWMLKRLLNLGS
ERRLKESIATVHEFAERIIRLRRKEKKSGDDLLSRFMAEGKNTDELLRDIVISFILAGRDTTSSALTWFFWLLSSNPDVE
QRILQEIREVRARRGLGDIKTEAFDLEELREMHYIHAAISEAMRLYPPVPVNTFMCSEDHVLPDGSSIKKGWFITYNAYA
MGRMEGIWGQECREFKPERWLAADGTFRPESPYRYPAFHAGPRMCLGKEMAYIQMKSIIACVLERFVVEVVDKDRKPEKT
LALTLRMKGGLLVTLRERCTAGVA

>KAJ0978714.1

MVLGALLVILATKFWQALFDLIWRPYIITKRFEKQGVRGPPYKFRMGAKQEMKDIQKGAETLVLDSRSHDIACKVLPHYQ
AWSSQYGTTFLYWMGTQPRICLGDPEMVKQVLSSKFGFYAKTYPGANIMAIMGKGLAFIEGSDWARHRRVINPAFHIDKL

DMMAKTMAECVLHTLSEWEKQGNDNKTQEIEVSRQFEELTCNIISHTVFGTSDSINGKKIFEAQKELFLITLADFDGSMR
YLPTKKNLKKWKLEKRMKNALTSIINSRLDNSKELGYGDDLLGLMLHSSFPVNGNNSSLSIDEIIDECKTFYFGGHETTS
QLLTWTMFLLSTNPEWQERLREEVLKECGTETPNTDMLSKLKLVTMVLWESLRLYPPAILLARDASKEMNLGGLMIPKGT
GLMIPMLMLHRDKEHWGEDANEFNPLRFENGASRAARHPNSILPFSIGPRACLGQNFAMLEARLVFAMMLQRSHDIACKV
LPHYQAWSSQYGTTFLYWMETKPRICLGDPEMVKQVLSSKFGFYAKPYPGANLMAMMGKGLVFTEGSDWARHRRVINPAF
HIDKLKMMAKSMADCVLNTLSEWEKQGNDNKTKEIEMSRQFQELTGNIISHAIFGTSDSINGKEIFEAQNELFLITIADL
NGSMRYLPTKKNLKKWKLEKRMKNTLTSIIKNRLDNSKELGYGDDLLGLMLHSSYSVNDNNSGLSIDEIIDECKTFYFAG
HETTSHLLTWTMFLLSTNPEWQERLREEVLKECGTETPNTDMLSKLKLVTMVLWESLRLYPPVILIARDASKDMNLAGLV
IPKGTGLMIPILMLQRDKEYWGEDANEFNPLRFENGASRAAMHPNSIVPFSIGPRACVGQNFAMLEARLVMAMILQRFSF
SLSPKYKHSPANWLTLQPQFGLPIDLRPLHQ

>KAJ0978746.1

MVLGALLVILATKLWQALFDLIWRPYIITKRFEKQGVRGPPYKFRMGAKQEMKDIQKGAETLVLDSRSHDIACKVLPHYQ
AWSSQYGTTFLYWMGTQPRICLGDPEMVKQVLSSKFGFYAKTYPGANIMAIMGKGLAFIEGSDWARHWRVINPAFHIDKL
NMMAKTMAECVLHTLSEWEKQGNDNKTLEIEVSRQFEELTCNIISHSVFGTSDSINGKKIFEAQKELFLITIADFNGSMR
YLPTKKNLKKWKLEKRMKNALTSIINSRLDNSKKLGYGDDLLGLMLHSSFPVNDNNSSLSIDEIIDECKTFYFGGHETTS
QLLTWTMFLLSTNPEWQERLREEVLKECGTETPNTDMLSKLKLVTMVLWESLRLYPPAILLARDASKDMNLGGLMIPKGT
GLMIPILMLQRDKEHWGEDANEFNPLRFENGASRAARHPNSILPFSIGPRACLGQNFAMLEARLVFAMMLQRFSFSLSPK
YKHSPVNWLTLQPQFGLPIDLRPLHQ

>KAJ0978747.1

MGYQLGMVLGALLVILATKLWQALFDLIWRPYIIIKRFEKQGVRGPPYKFRLGAKQEIQDIQKAAETLVLDSRSHDIACK
VLPHYQAWSSHYGTTFLYWMETKPRICLGDPEMVKQVLSSKFGFYAKPYPGANLMAMMGKGLVFTEGSDWARHRRVINPA
FHIDKLKMMAKSMADCVLNTLSEWEKQGNDNKTKEIEMSRQFQELTGNIISHAIFGTSDSINGKEIFEAQNELFLITIAD
LRGSMRYLPTKKNLKKWKLEKRMKNTLTSIIKNRLDNSKELGYGDDLLGLMLHSSYSVNDNNSGLSIDEIIDECKTFYFA
GHETTSHLLTWTMFLLSTNPEWQDRLREEVLKECGTETPNTDMLSKLKLVTMVLWESLRLYPPVILIARDASKDMNLGGL
MIPKGTGLMIPMLMLQRDKEYWGEDANEFNPLRFENGASRAAMHPNSILPFSIGPRACVGQNFAMLEARLVMAMILQRFS
FSLSPKYKHSPANRLTLQPQFGLPIDLRPLHQ

>KAJ0978942.1

MDHLSLPSILILFSLSIIFLFFSVSTQKKQSQPSNGLRPYPIVGDLPQFLRHRHRFLDWMTELLAASPTNTISFRRLGNV
RGVITANPSNVEHILKSNFENYPKGPRFIHHLQDFLGRGIFNVDGHLWRVQRKTASFEFNTRSLRNFVVVNVQHEILSRL
LPLLRKSSRTGAAIDLQDVLERFAFDNVCKVAFNEDPACLSDVSEKPLNLSSRFAQAFKRAAELSAGRFRYAIPRFWIIT
RLLNIGSERELRESIATVHGFATQIIRSRKKEKLRSDDLLSRFISNEDNSDEFLRDIVISFILAGRDTTSSALTWFFWLL
SRHPEVEHKILQEISSVRAARQLGDNTETFEFEELRQMQYLHAAISEAMRLYPPVSVNSTMCQSDDTLPDGTLLKKGMFM
SYNAYSMGRMESIWGKDCVEFKPERWLDGEGAFRPENPYRYPVFHAGPRMCLGKEMAYIQMKSIAACVLERFMVDVLHKD
KAPEQMLSLTLRMKHGLSVRIRDRN

>KAJ0980322.1

MEYSFLDLLSILGVFAFAFLCHKWIKNLTRTNTNVPPQASGALPVIGHLHLLRGPKPVFKLLSDMADKYGPAFTIRLGSV
RTLILSNWETTKDCFTTNDIVVAARPVNAATEYLGHNYAMFGFAPYGAYWRSVRKITLTDLLSPARLNMLKHVPAAEVDT
CMKELFTLCNSCDQNPNHVAKVDMKQWFGHLNYNIVVQMVAGKRYFGSGSASEEAWRFRDAATQFFHLLLSFVPSDMFPF
LAWMDIGGYVKAMKKASKEMDDVMDGLVEEHRKRRACGEVNEHPDFIDLMLSALEGEKLEGFDWKTVIKTTSLTLILGGT
DTTSTSLTKVLANLLNHREVLKKVQEELDEQVGRDRVVNESDTKKLVYLQAVIKESFRMTPPDPFLIRRATQKDCILAGY
HVPKGTQVLVNAWKVHNDPNVWPEPSEFRPERFLSSQKGIDVKGQHYELIPFGTGRRVCPGVTMALQVMNLTLARLIQGF
ELRTPGDVPVEIEGGLVSLASDSDPFMVELTPRLSPELY

>KAJ0981901.1

MSLLGKPTIVSADADFNRYILQSEGKMFENSCPTSIAEILGRWSMLALEGEIHREMRSIAVNFMGNVKLRNYFLPDIEQQ

ALKILHSWKDGSTFSAHEEGKKFAFNLMVKHLMSMDPGMPETVKLRKEYITFMKGMASIPLNLPYTAYRKALQSRSAILK
IMGKRLDERVQKIREGSHELEEDDLLASVSTQSNLTRDQILDLILSLLFAGHETSSAAISLAIYFLESSPKALQQLREEH
VNIVKMKGESGLTWEDYKQMEFTHCVINETLRLGNIVKFLHRKVIKDVQYKGYDIPCGWELVPIISAAHLDPTIYDDPQT
YNPWRWQTISTATSKNNNLMSFSGGPRLCPGAELAKLEMAVFLHHLVQKFNWELAEHDYPVSFPFLGFPKNLPIKVHAIK
KLT

>KAJ0981902.1(42)

METLTLSIAAVLLLLLASILWRLRHVQGSKTKNRSGSLFELMKNSHRILDWTTELLHDSPSGTTVTFMGVIISNPSNVEH
ILKNRFENYPKGSGFTSVLSDFLGSGIFNADGEHWRLQRKTASLEFSTKAIRAFIHSRVRLESVSRLIPLLSSAARSGEQ
LNLQDLLEFFTFDNACQVTFGHDPSLLSSPNDDDPLSSDRQFAKAFEEAAQLSVRRFSHPFPFIWKLQRFLNVGSEGRLK
EEVAKVQAFAMDVVQERKRKRDRTGSLGDDLLSRFIADNDYSDEFLRDIVISFVLAGRDTTAAAISWFFWLISTRPAVTD
LIVDEIRSVRAKCGKTMEQEPVFSLDQVRGMDYLHAALAESLRLYPPVPLQTRTCLEDDLLPDGTPVRKGQTVMYSSYAM
GRTERIWGQDWGEFRPERWLEKGVFRAVSPFTYPVFHAGPRMCLGKEMAHIQMKAVAAALLERFEIEVVDEAKEREKDLG
MMLRIKGGLPVRIRERMFLN

>KAJ0981907.1

MSPHMELLLLLSPVLLSIFFLSNLIKWRRRRRKLPPGGSGWPFIGETFAYLKPHKATSIGHFMKQHMTRYGKIYRSNLFG
EPTIVSADPGLNRFILQNEGRLFECSYPKSIGGILGKWSMLVLVGDMHRDMRMISLNFMSSGRLRLHLLPEVERHTLLVL
RAWKEGSSFSAQEEAKKFTFNLMAKNIMSMDPGEPETEKLRLEYITFMKGVVSAPLNFPGTPYWKALKSRSSILSVIERK
MDKRLLKLRDGDDTLEVDDLLSWALKQSNLSKEQILDLLLSLLFAGHETSSMALALAIFFLEACPKAVEELRVEHVEISR
KKKLRGEIGLNWDDYKQMEFSQCVINETLRLGNVVNFVHRKALKDVHYKGYEIPSGWKVLPVFAAVHLDPSLYSDPQEFN
PWRWQKSSSSNTNNFMPYGGGPRLCAGSELAKLEMAVFLHHLVLNYQWTLDEPDCPLAYPYIDFPKGLPIKVQRIT

>KAJ0982068.1

MSMGDLFFSIINVFSILLILILILKHYYRNPNPNPNPPSPPGLPLVGHLHLLKRQPLYRTLARLSDLYGPILLLRFGSRR
VLLVSSSSGAGECFTVNDIAFANRPAFLFGKHLGYNYSTLSWSPYGPHWRNLRRIATIEVLSTLRLLSSSHLRSDEVRCL
VKALLRDYAGPGFHLAEMKTKFFGLTYNVVMRMLANKKYYGEETEGSSEAGNEFRDIVKETFLIAGASNPADFVPVMRWL
GIGGHERRLKSLSRRRDNFFQALVNEHRDRKESGSQDGESSPAGRSTVIDLLLSMQEGDSEYYTDDVIKGFIAQMLVAGT
DTSAITMEWAMSLLLNNPQALKKLRVELDANISQGSLMQEVDLPKLPYLHSVINETLRMYPAGPLLVPHESSQDCTVGGF
HVPSGTILLVNAWKIQRDPELWDEPDKFKPERFLKEDVNEVFKMMPFGLGRRRCPGEGLAMRVVALVVGTLVQCFEWEKV
GDEDVDMSEGTGLTLPKAKPLVAMYKPREDMAAGLLSQL

>KAJ0985657.1

MEEMLELLWAWIWPLVWLSLSLVMVMKVLEVFYWRPKRIEKHFSRQGIKGPSYRFFIGNVKEMVSLIFEASSKPMMPCNS
HNILPRVLSFYHHWKKIYGSTFVLWFGPIARLTVADPDLIREIFVSRSEYFERYEAHPLVRQLEGDGLISLRGEKWAHHR
KVITPTFHMDNLKLLMPLIGKSMVEMVEKWSSVSSSGDFEIDVYDWFHSLTEDAISQTVFGRNYEDGKAVFQLQAQQMLF
AAEAFYKVLIPGYRYLPTRKNTISWKLEKEIRRKLVRLISRRKDDHDDEEKADDDGRAKDLLGLMISESRKRFPLRLSGP
SSTIISVQDIMQECKTFFFAGKQTTSSLLTWATVLLAMHSDWQELARQEVLSVCGSSDIPTRDHLSKLKTLGMVLYETLR
LYPPAVATIRSARADVDLGGYHIPRGTELLIPIIAVHHDTSLWGSDAAEFNPARFAGGVALAAKHPAAFIPFGLGARTCI
GQNLALLEAKLTVAILLQRFSFRLAPSYVHAPTVLMLLYPRYGSPVIFRRLSSNQHNHPST

>KAJ0985668.1

MEEMLELLWAWIWPLVWLSLSLVMVMKVLEVFYWRPKRIEKHFSRQGIKGPSYRFFIGNVKEMVSLIFEASSKPMMPCNS
HNILPRVLSFYHHWKKIYGSTFVLWFGPIARLTVADPDLIREIFVSRSEYFERYEAHPLVRQLEGDGLISLRGEKWAHHR
KVITPTFHMDNLKLLMPLIGKSMVEMVEKWSSVSSSGDFEIDVYDWFHSLTEDAISQTVFGRSYEDGKAVFQLQAQQMLF
AAEAFYKVLIPGYRYLPTRKNTISWKLEKEIRRKLVRLISRRKDDHDDEEKADDDGRAKDLLGLMISESRKRFPLRLSGP
SSTIISVQDIMEECKTFFFAGKQTTSSLLTWATVLLAMHCDWQVLARQEVLSVCGSSDIPTRDHLSKLKTLGMVLYETLR
LYPPAVATIRSARADVDLGGYHIPRGTELLIPIIAVHHDTSLWGSDAAEFNPARFAGGVALAAKHPAAFIPFGLGARTCI
GQNLALLEAKLTVAILLQRFVPAGAQLRPRAHRPHAPLPSIRFAGHLPPLVVKPTQSPFHVTAEMPSQSTLYFALLVFLL

FFKKI

>KAJ0985924.1(DzCYP72A1)

MESVMGVVWAAAAVAVVAWAWRTLDWVWWKPRRLDRELRRQGLRGNQYRLLHGDLKENARLSEKAKSRPLPLHCHDIAPR

VLPVLHNAIKDHGKISITWFGPYPRVTLMEPELVKEVLSNKFGHFAKIRATPLANFLVQGLVTYEGEKWAKHRRIINPAF

HLEKLKLMLPAFSTSCGELIRRWEKMIPDEGSQELKCLSRAPRPHKRCHLQDCIRSSYEEGRRIFELLAEQIQLLIPAFQ

TIYIPGYRFLPTPMNKRRSQVYNEMKRILKGMIEKREKAIRMGDSSKNDLLGLLLDSNMKEGEEHGKSQNKGMSTEDVIE

ECKLFYFAGQETTAVLLTWTMILLSMYPNWQAKAREEVLQVFGKNTPDMEGLSHLKIVTMILYEVLRLYPPFVLLRRKTY

KAMELGGIIYPPGVMLSLPLLFIHHDPAFWGEDAKEFNPERFSEGISKASKVPGAFFPFGGGPRICIGQSFAMIEAKIGI

CMILQCFSFELSPSYIHAPHTVITLQPQHGAQLMLQKL

>KAJ0985925.1(DzCYP72A2/9)

MESVMGVVWAAAAVAVAVVAWAWRTLDWVWWTPRRLDRELRQQGLRGNQYRVLHGDLKENARLSEEAKSRPLPLHCHDIA

PRVVPVLHNAIKDHGKISITWFGPYPRVTLMEPELVKEVLSNKFGHFAKVRPNPLTKLLVQGLVVYEGEKWAKHRRIINP

AFHLEKLKLMLPAFSTSCGELIRRWEKMIPDEGSHELNVFPELQDLTKDVISRTAFGSSYEEGRRIFELLAEQIQLLIPA

FQTIYIPGYRFLPTPMNKRRSQVYNEMKRILKGMIEKREKAIRMGDSSKNDLLGLLLDSNMKEGEEHGKSQNKGMSTEDV

IEECKLFYFAGQETTAVLLTWTMILLSMYPNWQAKAREEVLQVFGKSTPDMEGLNHLKIVTMILYEVLRLYPPFVFLTRK

TYKAMELGGITYPQGVILSLPLLFIHHDPAFWGEDAKEFNPERFSEGISKASKVPGAFFPFGGGPRICIGQSFAMIEAKI

GICMILQRFSFELSPSYIHAPHTVITLQPQHGAQLMLQKL

>KAJ0985926.1(DzCYP72A3)

MELVMGVVWAAVAVVAWAWRTLDWVWWTPRRLDRELRRQGLRGNQYRLLHGDLKENAQLSDEAKSRPLPLHCHDIAPRVL

PLFHNAIKDHGKISITWLGPYPRVILAEPELVKEVLSNKFGHFVKPSTTPLAKFLVQGLASYDGEKWVKHRRIINPAFHF

EKLKQMLPAFSTSCGELIRRWNKMIPDEGSQELNVFPELQGLTKDIISRTAFGSSYEEGRRIFELLTEQIKLTIPAFKTV

YIPGYRFLPTPMNKRRSQVYNEMKRILKGMIEKREKAIRMGESSKNDLLGLLLDSNMQEGEEHGKSQDKGMSTEDVIEEC

KLFYIAGQETTSALLTWTMILLSMYPNWQANAREEVLQVFGKSTPDIEGLSHLKIVTMILYEVLRLYPPGVFLDRKTYKA

MELGGITYPSGVILSLPLLFIHNDPTFWGEDAKEFNPERFSEGISKASKVPGAFFPFGGGPRICIGQNFALMEAKIGICM

IIQHFSFVLSPSYIHAPHSVITLQPQHGAQLMLQKL

>KAJ0985927.1(DzCYP72A4)

MELVMGVVWAAAVVMLVAWAWRTLDWVWRTPMRLDRELRRQGLRGNQYRVFHGDLKENARLSKEAESRPLPLHCHDIAPR

VLPLFHNAIKDHGKISITWLGPCPRVTLTEPELVKEVLNKFGHFVKPSTTPFAKFLVQGLVYYEGEHWAKHRRILNPAFH

LEKLKLMLPAFSTSCSELIRRWEKMIPDEGSQELNVFPELQGLTKDVISRTAFSSSYEEGRRIFELLKEQIQLYIQVYKT

VYIPGYRFLPTPMNKRRSQVYNEMKRILKGMIGEERKGHKNGESCKDDLLGLLLDSNMKEGEEHGKSQNKGMSTEEVIEE

CKLFYFAGQETTPSLLLTWTMILLSMYPNWQTKAREEVLKVFGKNTPDMEGLSHLKIVTMILYEVLRLYPPVVFITRKTY

KAMELGGITYPPGVIFSLPLLFIHHDPTFWGEDAKEFNPERFSEGISKASKVPAAFFPFGGGPRICIGQNFALIEAKIGI

CMILQHFSFVLSLLSYIHAPHTVITLQPEHGAQLMLQKL

>KAJ0985933.1(DzCYP72A5)

MESVMGVVWAAAAVAVVAWAWRTLDWVWWTPRRLDRELRQQGLRGNQYRVLHGDLKENTRLSEEAKSRPLPLHCHDIAPR

VVPVLHNAIKDHGKISITWFGPYPRVTLMEPELVKEVLSNKFGHFAKVRPNPLTKLLVQGLVVYEGEKWAKHRRIINPAF

HLEKLKLMLPAFSTSCGELIRRWEKMIPDEGSHELNVFPELQDLTKMSSPGLHSVAAMKKEGRRIFELLAEQIQLLIPAF

QTLYIPGYRFLPTPMNKRRSQVYNEMKRILKGMIEKREKAIRMGEGSKNDLLGLLLDSNIKESEEHGKSQNKVMSTEDVI

EECKLFYFAGQETTSLLLTWTMILLSMYPNWQAKAREEVLQVFGKNTPDMEGLSHLKIVTMILYEVLRLYPPFVLLRRKT

YKAMELGGITYPPGVILSLPLLFIHHDPAFWGEDAKEFNPERFSEGISKASKVPGAFFPFGGGPRICIGQSFAMIEAKIG

ICMILQCFSFELSPSYIHAPHTVITLQPQHGAQLMLQKL

>KAJ0985934.1(DzCYP72A6)

MELVMGVVWAAVAVVAWAWRTLDWVWWTPRRLDRELRRQGLRGNQYRVLHGDLKENAQLSEEAKSRPLPLHYHDIAPRVL

PLFHNAIKDHEPELVKEVLSNKFGHFVKPSTTPLAKFLVQGLASYDGEKWVKHRRIINPAFHFEKLKQMLPAFSTSCGEL

IRRWNKMIPDEGSQELNVFPELQGLTKDIISRTAFGSSYEEGRRIFELLTEQIKLTIPAFKTVVYIPGYRFLPTPMNKRRS
QVYNEMKRILKGMIEKREKAIRMGESSKNDLLGLLLDSNMQEGEEHVKSQDKGMSTEDVIEECKLFYIAGQETTSALLTW
TMILLSMYPNWQANAREEVLQVFGKSTPDIEGLSHLKIVTMILYEVLRLYPPGVFLDSKTSKAMELGGITYPSGVILSLP
LLFIHNDPTFWGEDAKEFNPERFSEGISKASKVTGAFFPFGGGPRICIGQNFALMEAKIGICMIIQHFSFVLSPSYIHAP
HSVITLQPQHGAQLMLQKL
>KAJ0985935.1(DzCYP72A7)
MRLDRELRRQGLRGNQYRVFHGDLKENARLSKEAESRPLPLHCHDIAPRVLPLFHNAIKDHGKISITWLGPCPRVTLTEP
ELVKEVLNKFGHFVKPSTTPFAKFLVQGLVSYEGEKWAKHRRILNPAFHLEKLNLMLPAFSTSCSELIRRWEKMIPDEGS
QELNVFPELQGLTKDVISRTAFSSSYEEGRRIFELLKEQIQLYIQVYKTVYIPGYRFLPTPMNKRRSQVYNEMKRILKGM
IEKREKAIRMGESCKDDLLGLLLDSNMKEGEEHGKSQNKGMSTEEVIEECKLFYFAGQETTSLLLTWTMILLSMYPNWQT
KAREEVLKVFGKNTPDMEGLSHLKIVTMILYEVLRLYPPVVFITRKTYKAMELGGITYPPGVIFSLPLLFIHHDPTFWGE
DAKEFNPERFSEGISKASKVPAAFFPFGGGPRICIGQNFALIEAKIGICMILQHFSFVLSLSYIHAPHTVITLQPEHGAQ
LMLQKL
>KAJ0985941.1(DzCYP72A8)
MESVMGVVWAAAAVAVVAWAWRTLDWVWWKPRRLDRELRRQGLRGNQYRLLHGDLKENARLSEEAKSRPLPLHCHDIAPR
VLPVLHNAIKDHGKISITWFGPYPRVTLMEPELVKEVLSNKFGHFAKIRATPLANFLVQGLVTYEGEKWAKHRRIINPAF
HLEKLKLMLPAFSTSCGELIRRWEKMIPDEGSQELNVFPELQDLTKDVISRTAFGSSYEEGRRIFELLAEQIQLLIPAFQ
TIYIPGYRFLPTPMNKRRSQVYNEMKRILKGMIEKREKAIRMGEGSKNDLLGLLLDSNIKESEEHGKSQNKVMSTEDVIE
ECKLFYFAGQETTSLLLTWTMILLSMYPNWQGKAREEVLQVFGKSTPDMEGLNHLKIVTMILYEVLRLYPPFVFLTRKTY
KAMELGGITYPQGVILSLPLLFIHHDPAFWGEDAKEFNPERFSEGISKASKVPGAFFPFGGGPRICIGQSFAMIEAKIGI
CMMLQRFSFELSPSYIHAPHTVITLQPQHGAQLMLQKL
>KAJ0985942.1(DzCYP72A2/9)
MESVMGVVWAAAAVAVAVVAWAWRTLDWVWWTPRRLDRELRQQGLRGNQYRVLHGDLKENARLSEEAKSRPLPLHCHDIA
PRVVPVLHNAIKDHGKISITWFGPYPRVTLMEPELVKEVLSNKFGHFAKVRPNPLTKLLVQGLVVYEGEKWAKHRRIINP
AFHLEKLKLMLPAFSTSCGELIRRWEKMIPDEGSHELNVFPELQDLTKDVISRTAFGSSYEEGRRIFELLAEQIQLLIPA
FQTIYIPGYRFLPTPMNKRRSQVYNEMKRILKGMIEKREKAIRMGDSSKNDLLGLLLDSNMKEGEEHGKSQNKGMSTEDV
IEECKLFYFAGQETTAVLLTWTMILLSMYPNWQGKAREEVLQVFGKSTPDMEGLNHLKIVTMILYEVLRLYPPFVFLTRK
TYKAMELGGITYPQGVILSLPLLFIHHDPAFWGEDAKEFNPERFSEGISKASKVPGAFFPFGGGPRICIGQSFAMIEAKI
GICMILQRFSFEL
>KAJ0985943.1(DzCYP72A10)
MELVMRVVWAVAAVAVVAWAWRTLDWVWWTPRRLDRELRRQGLRGNQYRLLHGDLKENAQLSEEAKSRPLPLHYHDIAPR
VLPLFHNAIKDHGKISITWLGPYPRVILTEPELVKEVLSNKFGHFVKPSTTPLAKFLVQGLASYDGEKWVKHRRIINPAF
HFEKLKQMLPAFSTSCGELIRRWNKMIPDEGSQELNVFPELQGLTKDIISRTAFGSSYEEGRRIFELLTEQIKLTIPAFK
TVYIPGYQFLPTPMNKRRSQVYNEMKRILKGMIEKREKAIRMGESSKNDLLGLLLDSNMQEGEEHGKSQDKGMSTEDVIE
ECKLFYIAGQETTSALLTWTMILLSMYPNWQANAREEVLQVFGKSTPDIEGLSHLKIVTMILYEVLRLYPPGVFLDRKTY
KAMELGGITYPSGVILSLPLLFIHNDPTFWGEDAKEFNPERFSEGISKASKVPGSFLPYGVGPRICIGQNFALMEAKIGI
CMIIQHFSFVLSPSYIHAPHSVITLQPQHGAQLMLQKL
>KAJ0985944.1(DzCYP72A11)
MELVMGVVWAAAVVMLVAWAWRTLDWVWRTPMRLDRELRRQGLRGNQYRVFHGDLKENARLSKEAESRPLPLHCHDIAPR
VLPLFHNAIKDHGKISITWLGPYPRVTLTEPELVIEVLNKFGHFVKPSTTPFAKFLVQGLVSYEGEKWAKHRRILNPAFH
LEKLKLMLPAFSTSCSELIRRWEEKIPDEGSQELNVFPELQGLTKDVISRTAFSSSYEEGRRIFELLKEQIQLYIQVYKT
VYIPGYRFLPTPMNKRRSQVYNEMKRILKGMIEKREKAIRMGESCKDDLLGLLLDSNMKEGEEHGKTQNKGMSTEEVIEE
CKLFYFAGQETTSLLLTWTMILLSMYPNWQAKAREEVLQVFGKNTPDMEGLSHLKIVTMILYEVLRLYPPVVFLTRKTYK
AMELGGITYPPGVIFSLPLLFIHHDPTFWGEDAKEFNPERFSEGISKASKVSAAFFPFGGGPRICIGQNFALIQAKIGIC

MILQHFSFVLSLSYIHAPHTVITLQPQHGAQLMLQKL

>KAJ0985945.1(DzCYP72A12)

MESLMGVIWAVAAVVVVAAAWRTLDWIWWTPRRLDRELRRQGLRGNQYRVLHGDLKENVRLSKEAKSRPLPLHCHDIAPR

VLPLFHNAIKDHGKISITWLGPYPRVTLTEPELVKEVLSNKFGHFVKPTTTPLAKFLVQGLVSYEGEKWAKHRRIINPAF

HLEKLKLMLPAFSTSSGELIRRWEKMIPNEGSQELNVFPELQDLTKDVISRTAFGSSYEDGRRIFELLTEQIQLLIPAFQ

TVYIPGFRFLPTPMNKRSSQVYNEMKRILIGMIEKREKAIRMGESNKNDLLGLLLDSNMKEGEEHRKSQNKGMSTEDVIE

ECKLFYFAGQETTSVLLTWTMILLSMYPNWQAKAREEVLQVFGKSAPDMEGLSHLKIVTMILYEVLRLYPPAVFLTRKTY

KAMELGGITYPPGVILSLPLLFIHHDPVFWGEDAKEFNPERFSEGISKASKVPGAFFPFGGGPRICIGQNFALIEAKIGI

CMILQHFSFVLSPSYIHAPHNVITLQPQHGAQLMLQKL

>KAJ0985946.1(DzCYP72A13)

MELVMGVIWTVTAAVVVVWAWRTLDWVWWTPRRLDRDLRRQGLRGNQYRLLHGDLKENARLSKEATSRPLPLHCHDIAPR

VAPLIHNAIKDHGKISITWLGPYPRVSLMDPDLVKEVLSNKFGHFVKPRITPIAKFLVQGLVAYEGEKWAKHRRIINPAF

HLEKLKLMLPAFSTSSGELIRRWEKMIPDEGSQELNVSPEIQNLTGDVISRTAFGSSYEEGRRIFELLTEQIHLTIPALQ

TVYIPGYRFLPTPMNNRRSQVYNEMKRILKGMIEKREKAIRMGESSKNDLLGLLLDSNMKEGEEQEKSQNKVMSTEDVIE

ECKLFYFAGQETTSVLLTWTMILLSMYPNWQAKAREEVLQVFGKSTPDMDGLSRLKIVTMILYEVLRLYPPVTFLTRKTY

KAMELGGINYPPGVLLQLPLLFIHHDPEFWGEDAKEFNPERFSEGILKASKVPGAFFPFGGGPRICIGQSFALIEAKIGI

SMILQHFSFVLSPSYIHAPHTALTLQPQHGAQLMLQKL

>KAJ0985950.1(DzCYP72A14)

MEYSVAAGVKEMIWGVATALLVVWVWRTLEWLWWKPRRLERELKRQGLRGGKYRLFHGDLKYNARLMKDALSRPLPPYSH

DVAPRVIPLVHQAIKIHGKMSITWLGPYPRVSLMDPELIREVLSNKFGHFVKPNFSPLVKLLAQGLASHEGEKWAKHRRI

INPAFHFEKLKCMLPAFSTCCDELVKRWQNKMNVEGSIELNMWPELQNLTGDVISRTAFGSCYEEGRRIFQLQTEQAELI

IPTAQTVYVPGFRFLPTPKNNRRKAIDREIKTILRSMIEKREKAIRMGGETSCKDDLLGLLLESNMKETEQQGRSKNKGL

TTEDVIEECKLFYLAGQETTSSLLTWTMVLLSMYPNWQAKAREEVLQVFGKNKPDMDGVGRLKIVTMILYEVLRLYPPAI

SLTRRTYKTMELGGITYPPGVLLLLPIILVHHDLDFWGEDAKEFKPDRFAEGISKASKVPGAFFPFGGGPRICIGQSFAL

IEAKLGLSMILQNFSFELSPSYIHAPYTLVTLQPQHGAPIKLHKL

>KAJ0987931.1

MGEGLVGWLMGLAVVVVVVVGLSLVMAMKVVDLLWWRPRRLEEHFSKQGIKGPPYRFFLGNVKEMVGFMLEASSKPMMPQ

NSHNILPRVLSFYHHWKKIYGSTFLLWFGPTPRLTVADPDLIRDILVSRSDSFDRYESHPLVRQLEGDGLVSLRGEKWAH

HRKVLTPTFHMDNLKLLIPYIGKTVLEMSDKLILAIPPSADEVEIDVSEWFQVVTEDAITRTAFGQSYDDGRAVCQLQAQ

QMLFAAEAFRKVLIPGYRFLPTKKNASSWRLDKEIKKNLVGLIKRRKDSSDDEERPDDGRPKDLLGQMINATTTAMSVHD

IVEECKTFFFAGKQTTSNLLTWTTVLLAMHPEWQDLARHEVLRVCGSRDIPTRDHLAQLKTLRMILNETLRLYPPAVATI

RRAKADVELGGYKIPRGTEILIPIMAVHHDAKIWGADAAEFNPGRFSNRAQNPTGFIPFGLGARMCIGKNLALLEAKLTI

AILLQRFSFRLAPRYVHAPTVLMLLYPQYGAPVIFRPLSPSIQSDDGPSESFL

>castasterone_C26-hydroxylase

MEGFCFQWLCVILFLYVLLLCIQKAFVHLWWTPKIIQKHFKKQGITGPKYHFLFGNLKEIASFTTPSWPS

TFTSHDILPNVLPFYHHWKKIYGSIFVIWFGPTARVTISDPALIRDIFVLKSDNFEKNESPALVKKLEGD

GLLSLKGEKWAHHRKIITPTFYIENLRLMIPMMGKSMKEMLDKWSKMSNASGKVEIEVSEMFSTLAEDVI

TRIVFGNSYEDGKAIFELQAQQMIYATEAYQKVFIPGYRFLPSKKNRICWRLDKQVRKSLMKLIEERRKK

EEVLSEECPNDLLEVMIKAGSDDEYRNTITVNDIVEECKTIFFAGKHTTSNLLTWTTILLAMHPKWQELA

RDEVLTVCGARDPPSKQQISKLKTLGMIINESVRLYPPAVAAIRRAKVDTQLGDFTLPRGTELLIPIIAI

HHDQTLWGQDANEFNPARFGLGVAQAAKHPMAFMPFGLGARRCVGQNLAVLQAKLAIAMILQRFSFDLSP

NYRHAPTILMLLCPQYGAPIIFQKL

>GhCYP734A1

MDGVLQWLKLVAVSFMVLVLVLKVIVLLWWRPKRIEDHFSRQGIRGPPYHFFIGNVKELVGMMLKASSQP

MPFSHNILPRVLSFYHHWKKIYGATFLVWFGPTVRLTVADPDLIREIFTSKSEFYEKNEAHPLIRQLEGD

GLLSLKGEKWAHHRKIITPTFHMENLKLLVPLVAQRVTHMLDKWSAMSTNTGEIEIEVCEWFQTLTEDVI

TRTAFGTSYEDGKAIFRLQAQQMVLAAEAFQKVFIPGYRFLPTKRNIRLWKLDRDVKKSLMKLIDGRKNK

LGNTVQEKGPKDLLGLMMQASNSSPNVTVHDIIEECKSFFFAGKQTTSNLLTWTTVLLAMHPHWQVLARE

EVLKVCGSRDIPSKDDVVKLKTLTMILNESLRLYPPTIATIRRAKIDAELGGYMILRDTELLIPILAVHH

DQAIWGNDANEFNPARFSEGVARAAKHPVGFIPFGLGVRTCIGQNLAILQAKLTLSIILQRFSFRLAPTY

QHAPTVLMLLYPQYGAPIIFQPLPESTVPRDQGS

>CYP734A1_11

MFSAMEEVWYWFKLLVICFMALVFVLKVVVLLWWRPRKIEHHFSKQGIRGPPYRFFIGNV

KELVGLMLKASSQPMPFSHNILPRVLSFYHHWKKIYGISSSFLLSSSLPLSFQFRFTFFT

TAVSSVCATFLVWFGPTVRLTISDPDLIREIFASKSEFYEKNEAHPLVKQLEGDGLLSLK

GEKWAHHRKIITPTLHMENLKLMVPVMAKSVTEMLEKWMAMSKSGEVEIEVSEWYQTLTE

DVITRMAFGSSYEDGKAIFQLQAQQMVMAAEAFQKVFIPGYRFLPTKRNMNSWKLDKEIK

KSLVKLIDRRKENRWKENPEKCPKDLLGLMIEETVKKGEMSWCPSSKITVQDIVEECKSF

FFAGKQTTSNLLTWTTVLLAMHPQWQVRARDEVFRVCGARDTPTKDDVVKLKTLSMILNE

SLRLYPPIIAAIRRAKTDVELGGYKIPRGMELLIPILAVHHDPLIWGNDANEFNPARFAE

GVARAAKHPVAFIPFGLGVRTCIGQNLAILQAKLALAIILQRFSFTLAPSYQHAPTVLML

LYPQYGAPITFRTLSTPDQGS

>AtCYP734A1

MEEESSSWFIPKVLVLSVILSLVIVKGMSLLWWRPRKIEEHFSKQGIRGPPYHFFIGNVK

ELVGMMLKASSHPMPFSHNILPRVLSFYHHWRKIYGATFLVWFGPTFRLTVADPDLIREI

FSKSEFYEKNEAHPLVKQLEGDGLLSLKGEKWAHHRKIISPTFHMENLKLLVPVVLKSVT

DMVDKWSDKLSENGEVEVDVYEWFQILTEDVISRTAFGSSYEDGRAVFRLQAQQMLLCAE

AFQKVFIPGYRFFPTRGNLKSWKLDKEIRKSLLKLIERRRQNAIDGEGEECKEPAAKDLL

GLMIQAKNVTVQDIVEECKSFFFAGKQTTSNLLTWTTILLSMHPEWQAKARDEVLRVCGS

RDVPTKDHVVKLKTLSMILNESLRLYPPIVATIRRAKSDVKLGGYKIPCGTELLIPIIAV

HHDQAIWGNDVNEFNPARFADGVPRAAKHPVGFIPFGLGVRTCIGQNLAILQAKLTLAVM

IQRFTFHLAPTYQHAPTVLMLLYPQHGAPITFRRLTNHED

>DzCYP94D143

MELTSLILVSSIILISTFLLLLQRRRTTRKQTIDGAPTLTFKDLVKNGHRFLDWTTE

MLLSSPTNTITLPSTVATSNPSNIEHILKYNFSNYPKGHNITDTLHDLLGDGIFNTDG

DHWKLQRKIASLQFNTKSIRSFVTNAVQIEVTTRLLPLLAAAAVSGEPIDLQDTLER

FSFDNVCKVAFDVDPARLAGDAMDGGRFARAFDTAAEISTNRFRQPRFFWLLRRK

LNLGEERRLKEAVRTVNEFAMKVVHDKKLKGKAGDDDLLSKFIEDDSEHSDEFLR

DIIISFVLAGRDTTSSALTWFFWLVSSRPEIRRAIRDEVSAVRAKHGSEPGQELKLEE

LREMEYLHAALSETLRLYPPVSLEPRACLADDKLPDGTEVKEGWSVMYNSYAMG

RMKSIWGEDCMEFRPERWLVNGEFQAKSPFKFPIFHAGPRTCLGKEMAYIQMKA

VAASVLERFELEMAPGEEKERAHGFTIVLRMNGGLPIVVKNRD

>PpCYP94D108

MDPPSLLSLALLLLAAAAAAAFVLFPRRRSPEKKPSSGNPGSLS

ELIKNGHRILDWMVEILAASPTNTVATYMGVVTANPANVEHMLKTKFENYPKGDRFVT

LLEDFLGRGIFNSDGDHWKLQRKTASLEFNTKTIRTFVMENVRVSVVDRLIPIFARAA

ASGETIDLQETLERFAFDNVCKVSFNEDTGRLSGDDTMEGREFARAFEQASELIVGRY

KHPFLLSWKLMRFFNIGDERRLKEKIATVHRFATSVIRRRKSAASLGDDLLSRFIAEA

DYPDEFLRDIIISFVLAGRDTTSATLTWFFWLASTRPEVLARVEAEVNAVRRKNGTCA

GVMFTLEEVREMDFLHAALSEALRLYPPVPLQTRACHESDEFPDGTKVRPGTTVMYNS

YAMGRMKSIWGEDYAEFRPERWLDKGGGFQPRSPFRFPVFHAGPRMCLGKEMAYIQMK

AVAASVVERFEVVVMDKEKVREKDYTMILRVKGGLPVRLKEKSVAAG

>PpCYP94D109

MESLSLIFISFITLIVFLVVSASKKRSHPSGYEPDIPKGGCPML

DSTAYLVLSSPTNTAVTSTGIITSNPENIEHVLKTNFANYPKGEHLTYGLYDLLGRGI

FNSDGDHWKLQRKIASLEFNTRTIRHFVTHDVSREVLDRLLPSLSRAANSGEIIDLQE

VLDRLAFDNVCKIAFDDDPARLADKKFNDGENDYYGKFAKAFGEAAEISTQRFEKSRW

KIARALNLGTERKMKNALAIVNEFAMQVVREMKRKRAEEGKGRGSSADLLSLFISEGE

FSDEFLRDVVISFVLAGRDTTSSTMAFFFWQVSTRPSICQKIKEEIVSVRKNHNNSQG

GAFTLEELREMDYLHAVLSETLRLYPIVPLHARYTLADDVLPDGTMVKKGSTVMHSIY

AMGRMESIWGADCLEFRPERWLENGVFRPKSPFLFPVFLAGPRMCLGKETAYMQMKAV

AASVMEKFKIEVADGKHESEREYYLEIVLRLKGGLPVRVTEKEWSDNSAVNV

>PpCYP72A616

MDSRVLGALAALLAAAAAWVMRAAAEWLWWRPRRLERSLRSQGV

DGNLYRFLNGDLKETVRLTKEARAQPISPPSHRFLHRINPLLLRAISHHGKFALTWIG

PTPRVSIMDPELVREVLSNKFGHFAKPKAAPIVKLLATGLANYEGEKWVRHRRIINPA

FHLEKLKRMLPAFFSCCSELIGRWESLVGCDESREVDVWPELQNLTGDVISRTAFGSS

YAEGRRIFQLQSEQAELLIQAVQTVYIPGYRFLPTPKNIRRTKIDKEVRALLRSIIEK

RENAMKMGDVHDDLLGLLMEYNLKESEHFNSKNIGMTTEDVIEECKLFYFAGQETTSV

LLTWTMILLGMHPSWQDRAREEVSQVFGKNKPDFDGLSRLKTVTMILYEVLRLYPPII

FLTRRTYKPMMLGGITFPPEVQLALPIIFIHHDPEFWGEDAEEFNPDRFADGVSKASK

NQMAFFPFGWGPRICIGQGFAMLEAKMGLSMILQRFSFELSPNYSHAPHTKITLQPQH

GAQMVLHRL

>TfCYP72A613

MVFLFPTGTIIIWVLTILLAVIPWYLLNKFWLKPKRFEKLLKAQ

GLQGDPYKLSALFMNNSKQDYILKLQQEAESKSIGLSKQAAPSIFSSHHQTVHKYGKN

SFLWEGTTPSVIITDPDQIKEVFDRIYDFPKQKLRSIAKYFSFGIIKYEGEKWAKHRK

IVNPAFHLDKLKGMLPAFSHSCNEMISKWKGLLSADGTCEVDVWPFLQNLTCDVISRT

AFGSSYAEGEKIFQLLKKQAFLLVTTLDKNIPLSLWWLLETTTKKRMKEIERDIRESL

EGIIEKREKALKNGETTNDDLLGILLQSNHAENQGHGNSKSIGMTTQEMIDECKLFYL

VGQETTSTLLVWTMVLLGRYPEWQARARQEVLQVFGNQNQNFEGLSQLKIVTMILYEV

LRLYPPAIYFNRALQKDLKLGNLSLPAGTLVSLPIILIHQDNDIWGDDAKEFKPERFA

EGIAKATKGQVSYFPFGWGPRICIGQNFALLEAKIAITSLLQNFSFELSPNYVHVPTT

VPFFQPKYGASIILHKL

>TfCYP82J17

MDSFLSQPITIVLAILSVLLYNIWKIRKPSNKFQKGMKLPQLSF

ALPLIGHLHLLGNQIPLAKTFASFADKYGPIFQIRLGAYPTLVISNKEAIKECFTTND

KILASRTKSTHGILLGYNHASFACAPYGRFWAKIRKVTMLELLSSRRVESLRHVYESE

IDTLVKDLSLYVKVGKVEVVISEWMERLTFNIITKMICGKRYFEYLQDVDDVEANGNI

VKLIKEVMHISGELVPKDVIPILGWFGFEGEVLKSMKRVSRDLDEVVGKWVEEHIEKS

DDGVNNSNEKQDLIDVMLSVIEDDPDSGNDRDTIIKANIVNLMIAGSDTTSTTMTWIL
VLLLNNMNALKRAQEEIDQHIGRDRKIESSDIKNLVYLQAIVKETLRLHPTLPLSIPH
EATEDCNIQGYYVPKGTRLFTNVWKLHRDPSIWLEPEKFSPERFINENGEIDHESHQF
EYLPFGLGRRACPGSMFATQVIHIAVARLIHLFDFEVPINEVVDMKQGTGLILSKFTP
LKVLLTPRLPYELYQ

>VcCYP94N1v1

MDLPSASAAVAAATAAVIFLLTIYLLPKKKSPASTGKNGSTSLESYPVIGNLPHFVKNRNRFLDWVAEII
SQSPTGTVIADPLVFTSNPENVEHTAKSRFDAYARGPAATAVLHDFLGSGILNVDGDSWRAQRKTASSEF
TTRSLRAFILDAVDGEAAGRLLPLLSRAAASGEVFDLQDVLERFAFDNICSIIFDADPNCLNDTHDGVGE
RFYHAFHDATLLSTGRYYYPFHWVWRLLRWLNLGTEKRLRDAVSDVHKAIDELVGSRKTEVGTTVRRQGG
GSDLLSRFAEGGDYSDDVLRDVLINFVLAGRDTTPSALTWFFFMISSRPDVVDQILDEIRSIRDHQDRSN
PNGGGGGFTLEELREMNYLHAAITESLRLNPPVPLMPKMCMEDDVLPDGTVVRRGWTVMYSAFAMGRKAE
IWGEDCMEFKPERCLDDGGCFKSASAYRLPAFHAGPRICLGKDMAYIQMKAVASSMLERFEVEVVEKRGK
PELSITMRMDRGLPVRIKERKRGC

>VcCYP94N1v2

MDLPSASAAVAAATAAVIFLLTIYLLPKKKSPASTGKNGSTSLESYPVIGNLPHFVKNRNRFLDWVAEII
SQSPTGTVIAAPLVFTSNPENVEHTAKSRFDAYARGPAATAVLHDFLGSGILNVDGDSWRAQRKTASSEF
TTRSLRAFILDAVDGEAAGRLLPLLSRAAASGEVFDLQDVLERFAFDNICSIIFDADPNCLNDTHDGVGE
RFYHAFHDATLLSTGRYYYPFHWVWRLLRWLNLGTEKRLRDAVSDVHKAIDELVGSRKTEVGTTVRRQGG
GSDLLSRFAEGGDYSDDVLRDVLINFVLAGRDTTPSALTWFFFMISSRPDVVDQILDEIRSIRDHQDRSN
PNGGGGGFTLEELREMNYLHAAITESLRLNPPVPLMPKMCMEDDVLPDGTVVRRGWTVMYSAFAMGRKAE
IWGEDCMEFKPERWLDDGGCFKSASAYRLPAFHAGPRICLGKDMAYIQMKAVASSLLERFEVEVVEKRGK
PELSITMRMDRGLPVRVKERKRGC
