# Supplementary material for: The Functional Characterization of DzCYP72A12-4 Related to Diosgenin Biosynthesis and Drought Adaptability in Dioscorea zingiberensis
Source: Int J Mol Sci. 2023 May 8;24(9):8430. doi: 10.3390/ijms24098430 (PMC10179397; doi:10.3390/ijms24098430)
Supplement: Supplementary file 1 [file ijms-24-08430-s001.zip › Table S3.pdf]

Table S3. Alignment of DzCYP72A nucleic acid sequences. Extreme similarity occurred in coding sequence of DzCYP72As belonging to Clade I (DzCYP1-13/23/24) and Clade II (DzCYP72A18-20), respectively. The first column in each paragraph contains gene or protein symbol, and the numbers in parentheses are sequence start location information. Completely consistent sequences are identified in yellow, and partially consistent sequences are identified in blue.

```
                       551                                                600
   DzCYP18     (551) AAGATGCTGGAGATGCAGCTGAGAAGACAGGGGTTGCCAGG-AACAAGTA
DzCYP72A17       (1) ---ATGCTGGAGATGCAGCTGAGAAGACAGGGGTTGCCCGGGAACAAGTA
DzCYP72A22       (1) ---ATGCTGGAGATGCAGCTGAGAAGACAGGGGTTGCCGGGGAACAAGTA
DzCYP72A20      (47) A-GATGCTGGAGATGCAGCTGAAAAGGCAGGGGTTGCCGGGGAACAAGTA
DzCYP72A21       (1) ---ATGCTGGAGATGCAGCTGAGAAGACAGGGGTTGCCCGGGAACAAGTA
                       601                                                650
   DzCYP18     (600) TCGGCTGATGATGGGGGACATGAAGGATGAGAAAAAGTCCTTCAAGGAGG
DzCYP72A17      (48) TCGGCTGATGATGGGGGACATGAAGGATGAGAAAAAGTCCTTCAAGGAGG
DzCYP72A22      (48) TCGGCTGATGATGGGGGACATGAAGGATGAGAAAAAGTCCTTCAAGGAGG
DzCYP72A20      (96) TCGGCTGATGATGGGGGACATGAAGGATGAGAAAAAGTCCTTCAAGGAGG
DzCYP72A21      (48) TCGGCTGATGATGGGGGACATGAAGGATGAGAAAAAGTCCTTCAAGGAGG
                       651                                                700
   DzCYP18     (650) CTTGGTCCAGGCCAATGGAGCTCACGCACAGGATAGCGGCTCGTGTGATT
DzCYP72A17      (98) CTTGGTCCAGGCCAATGGAGCTCACGCACAGGATAGCGGCTCGTGTGATT
DzCYP72A22      (98) CTTGGTCCAGGCCAATGGAGCTCACGCACAGGATAGCGGCTCGTGTGATT
DzCYP72A20     (146) CTTGGTCCAGGCCAATGGAGCTCACGCACAGGATAGCGGCTCGTGTGATT
DzCYP72A21      (98) CTTGGTCCAGGCCAATGGAGCTCACGCACAGGATAGCGGCTCGTGTGATT
                       701                                                750
   DzCYP18     (700) CCCTATGATCATCAAAT---GGCTCAAACACATGGCAAGATATCATTCAA
DzCYP72A17     (148) CCCTATGATCATCAAAT---GGCTCAAACACATGGCAAGATATCATTCAA
DzCYP72A22     (148) CCCTATGATCATCAAAT---GGCTCAAACACATGGCAAGATATCATTCAA
DzCYP72A20     (196) CCCTATGATCATCAAATAATGGCTCAAACACATGGCAAGATATCATTCAA
DzCYP72A21     (148) CCCTATGATCATCAAAT---GGCTCAAACACATGGCAAGATATCATTCAA
                       751                                                800
   DzCYP18     (747) ATGGAATGGAACAACTCCCAGAGTGAACATATGGAATCCAGAGATGTCGA
DzCYP72A17     (195) ATGGAATGGAACAACTCCCAGAGTGAACATCTGGAATCCAGAGATGTCGA
DzCYP72A22     (195) ATGGAATGGAACAACTCCCAGAGTGAACATCTGGAATCCAGAGATGTCGA
DzCYP72A20     (246) ATGGAAAGGGACAACTCCCAGAGTGAACATATGGAATCCAGAGATGTTGA
DzCYP72A21     (195) ATGGAATGGAACAACTCCCAGAGTGAACATCTGGAATCCAGAGATGTCGA
                       801                                                850
   DzCYP18     (797) GAGAGATCCTGTTAAACAAGTCTGGCCACATTATCAAGCCACAACTTAAC
DzCYP72A17     (245) GAGAGATCCTGTTGAACAAGTCCGGCCACATTATCAAGCCACAACTTAAC
DzCYP72A22     (245) GAGAGATCCTGTTGAACAAGTCTGGCCACATTATCAAGCCACAACTTAAC
DzCYP72A20     (296) AAGAGATCCTGTTGAACAAGTCTGGCCACATTATCAAGCCACAAGTGAAC
DzCYP72A21     (245) GAGAGATCCTGTTGAACAAGTCTGGCCACATTATCAAGCCACAACTTAAC
                       851                                                900
   DzCYP18     (847) CCTCTGATAAGGCTGCTTACAATGGGAGTATCTACACTTGAAGGAGAAGA
DzCYP72A17     (295) CCTCTGATAAGGCTGCTTACAATGGGAGTATCTACACTTGAAGGAGAAGA
DzCYP72A22     (295) CCTCTGATAAGGCTGCTTACAATGGGAGTATCTACACTTGAAGGAGAAGA
DzCYP72A20     (346) CCTCTGATAAAGCTGCTTACAATGGGAGTATCTACACTTGAAGGAGAAGA
DzCYP72A21     (295) CCTCTGATAAGGCTGCTTACAATGGGAGTATCTACACTTGAAGGAGAAGA
                       901                                                950
   DzCYP18     (897) ATGGGCACAGAGGAGGAAGTTGATCAATCCTGCCTTTCATATGGAGAAAC
DzCYP72A17     (345) ATGGGCACAGAGGAGGAAGTTGATCAATCCTGCCTTTCATATGGAGAAAC
DzCYP72A22     (345) ATGGGCACAGAGGAGGAAGTTGATCAATCCTGCCTTTCATATGGAGAAAC
DzCYP72A20     (396) ATGGGCACAGAGGAGGAAGTTGATCAATCCTGCCTTTCATATGGAGAAAC
DzCYP72A21     (345) ATGGGCACAGAGGAGGAAGTTGATCAATCCTGCCTTTCATATGGAGAAAC
                       951                                               1000
   DzCYP18     (947) TCAAGGAGATGGTACCTGCTTTTCGAATCAGTTGCATTGATTTAGTCAAG
```

```
DzCYP72A17   (395) TCAAGGAGATGGTACCTGTTTTTCGAATCAGTTGCATTGATTTAGTCAAG
DzCYP72A22   (395) TCAAGGAGATGGTACCTGTTTTTCGAATCAGTTGCATTGATTTAGTCAAG
DzCYP72A20   (446) TCAAGGAGATGGTACCTGCTTTTCGAATCAGTTGCATTGATTTAGTCAAG
DzCYP72A21   (395) TCAAGGAGATGGTACCTGTTTTTCGAATCAGTTGCATTGATTTAGTCAAG
                   1001                                          1050
   DzCYP18   (997) AGGTGGGAGAACTT---AGTGAGTGCCGAAGGATCCTGTGAACTAGATGT
DzCYP72A17   (445) AGGTGGGAGAACTT---AGTGAGTGCCGAAGGATCCTGTGAACTAGATGT
DzCYP72A22   (445) AGGTGGGAGAACTT---AGTGAGTGCCGAAGGATCCTGTGAACTAGATGT
DzCYP72A20   (496) AGGTGGGAGAACTT---AGTGAGTGCCGAAGGATCCTGTGAACTAGATGT
DzCYP72A21   (445) AGGTGGGAGAACTTGGTAGTGAGTGCCGAAGGATCCTGTGAACTAGATGT
                   1051                                          1100
   DzCYP18  (1044) CTGGCCTGAATTTCAGAGTCTAACAGGAGATGTGATCTCTCGCACTGCAT
DzCYP72A17   (492) CTGGCCTGAATTTCAGAGTCTAACAGGAGATGTGATCTCTCGCACTGCAT
DzCYP72A22   (492) CTGGCCTGAATTTCAGAGTCTAACAGGAGATGTGATCTCTCGCACTGCAT
DzCYP72A20   (543) CTGGCCTGAATTTCAGAGTCTAACAGGAGATGTGATCTCTCGCACTGCAT
DzCYP72A21   (495) CTGGCCTGAATTTCAGAGTCTAACAGGAGATGTGATCTCTCGCACTGCAT
                   1101                                          1150
   DzCYP18  (1094) TTGGTAGTAGTTTTGAGGAAGGAAAGCAGATTTTTGAGTTCCAAAAGGAA
DzCYP72A17   (542) TTGGTAGTAGTTTTGAGGAAGGAAAGCAGATTTTTGAACTCCAAAAGGAA
DzCYP72A22   (542) TTGGTAGTAGTTTTGAGGAAGGAAAGCAGATTTTTGAACTCCAAAAGGAA
DzCYP72A20   (593) TTGGTAGTAGTTTTGAGGAAGGAAAGCAGATTTTTGAACTCCAAAAGGAA
DzCYP72A21   (545) TTGGTAGTAGTTTTGAGGAAGGAAAGCAGATTTTTGAACTCCAAAAGGAA
                   1151                                          1200
   DzCYP18  (1144) CAAGCTGTTCTTGTCATTGAAGCTGCCCGCTCGATCTATCTTCCAGGCTT
DzCYP72A17   (592) CAAGCTGTTCTTGTCATTGAAGCTTCCCGCTCGATCTATCTTCCAGGCTT
DzCYP72A22   (592) CAAGCTGTTCTTGTCATTGAAGCTTCCCGCTCGATCTATCTTCCAGGCTT
DzCYP72A20   (643) CAAGCTGTTCTTGTCATTGAAGCTGCCCGCTCGATCTATCTTCCAGGCTT
DzCYP72A21   (595) CAAGCTGTTCTTGTCATTGAAGCTGCCCGCTCGATCTATCTTCCAGGCTT
                   1201                                          1250
   DzCYP18  (1194) TAGGTTCCTGCCAACTGCAAAGAATAAGAGGAGAATGTTCATCGACAGTG
DzCYP72A17   (642) TAGGTTCCTGCCAACTGCAAAGAATAAGAGGAGAATGTTCATCGACAGTG
DzCYP72A22   (642) TAGATTCCTGCCAACTGCAAAGAATAAGAGGAGAATGTTCATCGACAGTG
DzCYP72A20   (693) TAGGTTCCTGCCAACTGCAAAGAATAAGAGGAGAATGTTCATCGACAGTG
DzCYP72A21   (645) TAGGTTCCTGCCAACTGCAAAGAATAAGAGGAGAATGTTCATCGACAGTG
                   1251                                          1300
   DzCYP18  (1244) AGATCAAGAGAATGCTACGAGATATCATCCACAAGAAGATAGATTCAATG
DzCYP72A17   (692) AGATCAAAAGAATGCTACGAGATATCATCCACAAGAAGTTAGATTCAATG
DzCYP72A22   (692) AGATCAAAAGAATGCTACGAGATATCATCCACAAGAAGTTAGATTCAATG
DzCYP72A20   (743) AGATCAAGAGAATGCTACGAGATATCATCCACAAGAAGATAGATTCAATG
DzCYP72A21   (695) AGATCAAGAGAATGCTACGAGATATCATCCACAAGAAGATAGATTCAATG
                   1301                                          1350
   DzCYP18  (1294) AAAATCGGAGAAAACACTGATGATGACTTACTCAGCTTGTTTATTTGCAA
DzCYP72A17   (742) AAAATCGGAGAAAACGCTGATGATGACTTACTCAGCTTGTT--GTTGCAA
DzCYP72A22   (742) AAAATCGGAGAAAACGCTGATGATGACTTACTCAGCTTGTT--GTTGCAA
DzCYP72A20   (793) AAAATCGGAGAAAATACTGATGATGACTTACTCAGCTTGTT--GTTGCAA
DzCYP72A21   (745) AAAATCGGAGAAAACACTGATGATGACTTACTCAGCTTGTT--GTTGCAA
                   1351                                          1400
   DzCYP18  (1344) TCCGATACTATGA-------------------------------------
DzCYP72A17   (790) TCCGATACGATGAATGTTGTCGCTGAAGATAAAAACAAGAAGAACAATGG
DzCYP72A22   (790) TCCAATACGATGAATGTTGCCGCTGAAGATAAAAACAAGAAGAACAATGG
DzCYP72A20   (841) TCCGATACGATGAATGTTGCCACTGAAGATAAAAACAAGAAAAACAATGG
DzCYP72A21   (793) TCCGATACTATGAATGTTGCCACTGAAGATAAAAACAAGAAAAACAATGG
                   1401                                          1450
   DzCYP18  (1357) --------------------------------------------------
DzCYP72A17   (840) GATCACGATTGATGATGTAATAGAGGAATGCAAGTTGTTCTACTTCGCTG
DzCYP72A22   (840) GATCACGATTGATGATGTAATAGAGGAATGCAAGTTGTTCTACTTCGCTG
DzCYP72A20   (891) GATCACGATTGATGATGTAATAGAGGAATGCAAGTTGTTCTACTTCGCTG
```

```
DzCYP72A21   (843) GATCACGATTGATGATGTAATAGAGGAATGCAAGTTGTTCTACTTTGCTG
                   1451                                              1500
   DzCYP18  (1357) --------------------------------------------------
DzCYP72A17   (890) GCCAAGAGGGCACCTCAATTTTGCTTACCTGGACATTGATTCTCTTATCC
DzCYP72A22   (890) GCCAAGAGGGCACCTCAATTTTGCTTACCTGGACATTGATTCTCTTATCC
DzCYP72A20   (941) GCCAAGAGACCACCTCAATTTTGCTTACCTGGACATTGATTCTCTTATCC
DzCYP72A21   (893) GCCAAGAGACCACCTCAATTTTGCTTACCTGGACATTGATTCTCTTATCC
                   1501                                              1550
   DzCYP18  (1357) --------------------------------------------------
DzCYP72A17   (940) ATGTACCCTAGTTGGCAGAAGAAAGCAAGGGAGGAAGTTCTCAATAACTG
DzCYP72A22   (940) ATGTACCCTAGTTGGCAGAAGAAAGCAAGGGAGGAAGTTCTCAATAACTG
DzCYP72A20   (991) ATGTACCCTACTTGGCAGAAGAAAGCTAGGGAGGAAGTTCTCAATACCTG
DzCYP72A21   (943) ATGTACCCTACTTGGCAGAAGAAAGCTAGGGAGGAAGTTCTCAATACCTT
                   1551                                              1600
   DzCYP18  (1357) --------------------------------------------------
DzCYP72A17   (990) CGGGAAGAACACACCTGAGTTCGAGAACATCAGCCACCTCAAGATTGTAA
DzCYP72A22   (990) CAGGAAGAACACACCTGAGTTCGAGAACATCAGCCACCTCAAAATTGTAA
DzCYP72A20  (1041) TGGGAAAAACACACCTGAGTTCGAGAACATCAGCCACCTCAAGATTGTAA
DzCYP72A21   (993) TGGGAAGAACACACCTGAGTTCGAGAACATCAGCCACCTCAAGATTGTAA
                   1601                                              1650
   DzCYP18  (1357) --------------------------------------------------
DzCYP72A17  (1040) ACATGATATTACATGAAGTATTGAGGTTGTATCCACCTGGGGTCACACTG
DzCYP72A22  (1040) ACATGATATTACATGAAGTATTGAGGTTGTATCCACCTGGGGTCACACTG
DzCYP72A20  (1091) ACATGATATTACATGAAGTATTGAGGTTGTATCCACCTGTGATCACACTG
DzCYP72A21  (1043) ACATGATATTACATGAAGTATTGAGGTTGTATCCACCTGTGATCACACTG
                   1651                                              1700
   DzCYP18  (1357) --------------------------------------------------
DzCYP72A17  (1090) ATTCGTTACATAAACAAGAAAGTCAAAGTGGGAAATATAACATTACCTGC
DzCYP72A22  (1090) ATTCGTTACATAAACAAGAAAGTCAAAGTGGGAAATATAACATTACCTGC
DzCYP72A20  (1141) TTTCGTCACATAAACAAGAATGTCAAACTGGGAGATATAACATTACCTGC
DzCYP72A21  (1093) TTTCGTCACATAAACAAGAATGTCAAACTGGGAGATATAACATTACCTGC
                   1701                                              1750
   DzCYP18  (1357) --------------------------------------------------
DzCYP72A17  (1140) TGGAGCTGAAGTCTTGATACCTATTCTACAAGTACACCATGATCCAGAAA
DzCYP72A22  (1140) TGGAGCTGAAGTCTTGATACCTATTCTACAAGTACACCATGATCCAGAAA
DzCYP72A20  (1191) TGGAGCTGAAGTCTTGATACCTATTCTACAAGTACACCATGATCCAGAAA
DzCYP72A21  (1143) TGGAGCTGAAGTCTTGATACCTATTCTACAAGTACACCATGATCCAGAAA
                   1751                                              1800
   DzCYP18  (1357) --------------------------------------------------
DzCYP72A17  (1190) TTTGGGGAGAAGATGCTGAAGAGTTCAAACCAGAGAGATTTTCAGAAGGG
DzCYP72A22  (1190) TTTGGGGAGAAGATGCTGAAGAGTTCAAACCAGAGAGATTTTCTGAAGGG
DzCYP72A20  (1241) TTTGGGGGAGGATGCTGAAGAGTTCAAAACCAGAGAGATTTTCAGAAGGG
DzCYP72A21  (1193) TTTGGGGGGAGGATGCTGAAGAGTTCAAACCAGAGAGATTTTCAGAAGGG
                   1801                                              1850
   DzCYP18  (1357) --------------------------------------------------
DzCYP72A17  (1240) GTTTCAAATGCATCAAAGGGTCAGCAAGCATTCTTTCCTTTTGGTTGGGG
DzCYP72A22  (1240) GTTTCAAATGCATCAAAGGGTCAGCAAGCATTCTTTCCTTTTGGTTGGGG
DzCYP72A20  (1291) GTTTCAAATGCATCAAAGGGTCAGAATGCATTCTTTCCTTTTGGTTGGGG
DzCYP72A21  (1243) GTTTCAAATGCATCAAAGGGTCAGAATGCATTCTTTCCTTTTGGTTGGGG
                   1851                                              1900
   DzCYP18  (1357) --------------------------------------------------
DzCYP72A17  (1290) CCCCAGAATATGTAGTGGGCAAACTTTCGCTATGATAGAAGCAAAGCTAG
DzCYP72A22  (1290) CCCCAGAATATGTAGTGGGCAAACTTTCGCTATGATAGAAGCAAAGCTAG
DzCYP72A20  (1341) CCCAAGAATATGTATTGGGCAAACTTTCGCTATGATAGAAGCAAAGCTAG
DzCYP72A21  (1293) CCCAAGAATATGTATTGGGCAAACTTTCGCTATGATAGAAGCAAAGCTAG
                   1901                                              1950
   DzCYP18  (1357) --------------------------------------------------
```

```
DzCYP72A17  (1340) CTCTGGCAATGGTGCTTCAAAATTTCTCCTTTGAACTCTCACCTTCCTAT
DzCYP72A22  (1340) CTCTGGCAATGGTGCTTCAAAATTTCTCCTTTGAACTCTCACCTTCCTAT
DzCYP72A20  (1391) CTCTGGCAATGGTGCTTCAACATTTCTCCTTTGACCTCTCTCCTTCCTAT
DzCYP72A21  (1343) CTCTGGCAATGGTGCTTCAACATTTCTCCTTTGACCTCTCTCCTTCCTAT
                   1951                                           2000
   DzCYP18  (1357) --------------------------------------------------
DzCYP72A17  (1390) ACTCATGCTCCTTACAATGTGATAACCCTTCAGCCACAATATGGAGCTCA
DzCYP72A22  (1390) ACTCATGCTCCTTACAATGTGATGACCCTTCAGCCACAATATGGAGCTCA
DzCYP72A20  (1441) ACTCATGCTCCTTACACTGTAATAACCCTTCAGCCACAATATGGAGCTCA
DzCYP72A21  (1393) ACTCATGCTCCTTACACTGTAATAACCCTTCAGCCACAATATGGAGCTCA
                   2001               2022
   DzCYP18  (1357) ----------------------
DzCYP72A17  (1440) TCTCATCCTACATCAACTCTGA
DzCYP72A22  (1440) TCTAATCCTACATCAACTCTGA
DzCYP72A20  (1491) TCTCATCCTACATCAACTCTGA
DzCYP72A21  (1443) TCTCATCCTACATCAACTCTGA


                  151                                              200
DzCYP72A17    (1) ----------------------------------MLEMQLRRQGLPGNKY
DzCYP72A22    (1) ----------------------------------MLEMQLRRQGLPGNKY
DzCYP72A20    (1) ------------------MGVLWRVLYLVWWKPKMLEMQLKRQGLPGNKY
DzCYP72A18  (151) ASKGQNAFFPFGWGPRILIGVLGRVSYLVWWKPKDAGDAAEKTGVARNKY
DzCYP72A19    (1) --------------------------------------------------
DzCYP72A21    (1) ----------------------------------MLEMQLRRQGLPGNKY
                  201                                              250
DzCYP72A17   (17) RLMMGDMKDEKKSFKEAWSRPMELTHRIAARVIPYDHQ-MAQTHGKISFK
DzCYP72A22   (17) RLMMGDMKDEKKSFKEAWSRPMELTHRIAARVIPYDHQ-MAQTHGKISFK
DzCYP72A20   (33) RLMMGDMKDEKKSFKEAWSRPMELTHRIAARVIPYDHQIMAQTHGKISFK
DzCYP72A18  (201) RLMMGDMKDEKKSFKEAWSRPMELTHRIAARVIPYDHQ-MAQTHGKISFK
DzCYP72A19    (1) --------------------------------------------------
DzCYP72A21   (17) RLMMGDMKDEKKSFKEAWSRPMELTHRIAARVIPYDHQ-MAQTHGKISFK
                  251                                              300
DzCYP72A17   (66) WNGTTPRVNIWNPEMSREILLNKSGHIIKPQLNPLIRLLTMGVSTLEGEE
DzCYP72A22   (66) WNGTTPRVNIWNPEMSREILLNKSGHIIKPQLNPLIRLLTMGVSTLEGEE
DzCYP72A20   (83) WKGTTPRVNIWNPEMLKEILLNKSGHIIKPQVNPLIKLLTMGVSTLEGEE
DzCYP72A18  (250) WNGTTPRVNIWNPEMSREILLNKSGHIIKPQLNPLIRLLTMGVSTLEGEE
DzCYP72A19    (1) --------------------------------------------------
DzCYP72A21   (66) WNGTTPRVNIWNPEMSREILLNKSGHIIKPQLNPLIRLLTMGVSTLEGEE
                  301                                              350
DzCYP72A17  (116) WAQRRKLINPAFHMEKLKEMVPVFRISCIDLVKRWENLVS-AEGSCELDV
DzCYP72A22  (116) WAQRRKLINPAFHMEKLKEMVPVFRISCIDLVKRWENLVS-AEGSCELDV
DzCYP72A20  (133) WAQRRKLINPAFHMEKLKEMVPAFRISCIDLVKRWENLVS-AEGSCELDV
DzCYP72A18  (300) WAQRRKLINPAFHMEKLKEMVPAFRISCIDLVKRWENLVS-AEGSCELDV
DzCYP72A19    (1) --------------------------------------------------
DzCYP72A21  (116) WAQRRKLINPAFHMEKLKEMVPVFRISCIDLVKRWENLVVSAEGSCELDV
                  351                                              400
DzCYP72A17  (165) WPEFQSLTGDVISRTAFGSSFEEGKQIFELQKEQAVLVIEASRSIYLPGF
DzCYP72A22  (165) WPEFQSLTGDVISRTAFGSSFEEGKQIFELQKEQAVLVIEASRSIYLPGF
DzCYP72A20  (182) WPEFQSLTGDVISRTAFGSSFEEGKQIFELQKEQAVLVIEAARSIYLPGF
DzCYP72A18  (349) WPEFQSLTGDVISRTAFGSSFEEGKQIFEFQKEQAVLVIEAARSIYLPGF
DzCYP72A19    (1) --------------------------------------------------
DzCYP72A21  (166) WPEFQSLTGDVISRTAFGSSFEEGKQIFELQKEQAVLVIEAARSIYLPGF
                  401                                              450
DzCYP72A17  (215) RFLPTAKNKRRMFIDSEIKRMLRDIIHKKLDSMKIGENADDDLLSLLLQS
DzCYP72A22  (215) RFLPTAKNKRRMFIDSEIKRMLRDIIHKKLDSMKIGENADDDLLSLLLQS
DzCYP72A20  (232) RFLPTAKNKRRMFIDSEIKRMLRDIIHKKIDSMKIGENTDDDLLSLLLQS
```

```
DzCYP72A18  (399) RFLPTAKNKRRMFIDSEIKRMLRDIIHKKIDSMKIGENTDDDLLSLFICN
DzCYP72A19    (1) --------------------------------------------------
DzCYP72A21  (216) RFLPTAKNKRRMFIDSEIKRMLRDIIHKKIDSMKIGENTDDDLLSLLLQS
                  451                                            500
DzCYP72A17  (265) DTMNVVAEDKNKKNNGITIDDVIEECKLFYFAGQEGTSILLTWTLILLSM
DzCYP72A22  (265) NTMNVAAEDKNKKNNGITIDDVIEECKLFYFAGQEGTSILLTWTLILLSM
DzCYP72A20  (282) DTMNVATEDKNKKNNGITIDDVIEECKLFYFAGQETTSILLTWTLILLSM
DzCYP72A18  (449) PIL-----------------------------------------------
DzCYP72A19    (1) -------------------------------------------------M
DzCYP72A21  (266) DTMNVATEDKNKKNNGITIDDVIEECKLFYFAGQETTSILLTWTLILLSM
                  501                                            550
DzCYP72A17  (315) YPSWQKKAREEVLNNCGKNTPEFENISHLKIVNMILHEVLRLYPPGVTLI
DzCYP72A22  (315) YPSWQKKAREEVLNNCRKNTPEFENISHLKIVNMILHEVLRLYPPGVTLI
DzCYP72A20  (332) YPTWQKKAREEVLNTCGKNTPEFENISHLKIVNMILHEVLRLYPPVITLF
DzCYP72A18  (452) --------------------------------------------------
DzCYP72A19    (2) YPTWQKKAREEVLNTCGKNTPEFENVSHLKIVNMILHEVLRLYPPVITLF
DzCYP72A21  (316) YPTWQKKAREEVLNTFGKNTPEFENISHLKIVNMILHEVLRLYPPVITLF
                  551                                            600
DzCYP72A17  (365) RYINKKVKVGNITLPAGAEVLIPILQVHHDPEIWGEDAEEFKPERFSEGV
DzCYP72A22  (365) RYINKKVKVGNITLPAGAEVLIPILQVHHDPEIWGEDAEEFKPERFSEGV
DzCYP72A20  (382) RHINKNVKLGDITLPAGAEVLIPILQVHHDPEIWGRMLKSSKPERFSEGV
DzCYP72A18  (452) --------------------------------------------------
DzCYP72A19   (52) RHINKNVKLGDITLPAGAEVVIPILQVHHDPEIWGEDAEEFKPERFSEGV
DzCYP72A21  (366) RHINKNVKLGDITLPAGAEVLIPILQVHHDPEIWGEDAEEFKPERFSEGV
                  601                                            650
DzCYP72A17  (415) SNASKGQQAFFPFGWGPRICSGQTFAMIEAKLALAMVLQNFSFELSPSYT
DzCYP72A22  (415) SNASKGQQAFFPFGWGPRICSGQTFAMIEAKLALAMVLQNFSFELSPSYT
DzCYP72A20  (432) SNASKGQNAFFPFGWGPRICIGQTFAMIEAKLALAMVLQHFSFDLSPSYT
DzCYP72A18  (452) --------------------------------------------------
DzCYP72A19  (102) SNASKGQNAFFPFGWGPRICIGQTFAMIEAKLALAMVLQHFSFDLSPSYT
DzCYP72A21  (416) SNASKGQNAFFPFGWGPRICIGQTFAMIEAKLALAMVLQHFSFDLSPSYT
                  651                  672
DzCYP72A17  (465) HAPYNVITLQPQYGAHLILHQL
DzCYP72A22  (465) HAPYNVMTLQPQYGAHLILHQL
DzCYP72A20  (482) HAPYTVITLQPQYGAHLILHQL
DzCYP72A18  (452) ----------------------
DzCYP72A19  (152) HAPYTVITLQPQYGAHLILHQL
DzCYP72A21  (466) HAPYTVITLQPQYGAHLILHQL
```

```
                  1                                               50
DzCYP72A1     (1) ATGGAGTCAGTGATGGGAGTGGTATGGGCGGCGGCGGCGGTGGCGGTG--
DzCYP72A8     (1) ATGGAGTCAGTGATGGGAGTGGTATGGGCGGCGGCGGCGGTGGCGGTG--
DzCYP72A2     (1) ATGGAGTCAGTGATGGGAGTGGTATGGGCGGCGGCGGCGGTGGCGGTGGC
DzCYP72A9     (1) ATGGAGTCAGTGATGGGAGTGGTATGGGCGGCGGCGGCGGTGGCGGTGGC
DzCYP72A5     (1) ATGGAGTCAGTGATGGGAGTGGTATGGGCGGCGGCGGCGGTGGCGGTG--
DzCYP72A10    (1) ATGGAGTTAGTGATGAGAGTGGTATGGGCGGTGGCGGCGGTGGCGGTG--
DzCYP72A3     (1) ATGGAGTTAGTGATGGGAGTGGTATGGGCGG------CGGTGGCGGTG--
DzCYP72A6     (1) ATGGAGTTAGTGATGGGAGTGGTATGGGCGG------CGGTGGCGGTG--
DzCYP72A11    (1) ATGGAGTTAGTGATGGGAGTGGTATGGGCGGCGGCGGTGGTGATGCTG--
DzCYP72A4     (1) ATGGAGTTAGTGATGGGAGTGGTATGGGCGGCGGCGGTGGTGATGCTG--
DzCYP72A7     (1) --------------------------------------------------
DzCYP72A12    (1) ATGGAGTCACTGATGGGAGTGATATGGGCGGTGGCGGCGGTGGTGGTG--
```

```
DzCYP72A13     (1) ATGGAGTTAGTGATGGGAGTGATATGGACGGTGACGGCGGCGGTGGTG--
                   51                                               100
 DzCYP72A1    (49) ----GTGGCGTGGGCGTGGAGGACGTTGGATTGGGTTTGGTGGAAGCCGA
 DzCYP72A8    (49) ----GTGGCGTGGGCGTGGAGGACGTTGGATTGGGTTTGGTGGAAGCCGA
 DzCYP72A2    (51) GGTGGTGGCGTGGGCGTGGAGGACGTTGGATTGGGTTTGGTGGACGCCGA
 DzCYP72A9    (51) GGTGGTGGCGTGGGCGTGGAGGACGTTGGATTGGGTTTGGTGGACGCCGA
 DzCYP72A5    (49) ----GTGGCGTGGGCGTGGAGGACGTTGGATTGGGTTTGGTGGACGCCGA
DzCYP72A10    (49) ----GTGGCGTGGGCGTGGAGGACGTTGGATTGGGTTTGGTGGACGCCGA
 DzCYP72A3    (43) ----GTGGCGTGGGCGTGGAGGACGTTGGATTGGGTTTGGTGGACGCCGA
 DzCYP72A6    (43) ----GTGGCGTGGGCGTGGAGGACGTTGGATTGGGTTTGGTGGACGCCGA
DzCYP72A11    (49) ----GTGGCGTGGGCGTGGAGGACGTTGGATTGGGTTTGGCGGACGCCGA
 DzCYP72A4    (49) ----GTGGCGTGGGCGTGGAGGACGTTGGATTGGGTTTGGCGGACGCCGA
 DzCYP72A7     (1) -------------------------------------------------A
DzCYP72A12    (49) ----GTGGCGGCCGCGTGGAGGACGTTGGATTGGATTTGGTGGACGCCGA
DzCYP72A13    (49) ----GTGGTGTGGGCGTGGAGGACGTTGGATTGGGTTTGGTGGACGCCGA
                   101                                              150
 DzCYP72A1    (95) GGAGGCTGGATCGGGAACTCCGGCGGCAGGGCCTGCGCGGCAACCAGTAC
 DzCYP72A8    (95) GGAGGCTGGATCGGGAACTCCGGCGGCAGGGCCTGCGCGGCAACCAGTAC
 DzCYP72A2   (101) GGAGGCTGGATCGGGAGCTCCGGCAGCAGGGCCTGCGCGGCAACCAGTAC
 DzCYP72A9   (101) GGAGGCTGGATCGGGAGCTCCGGCAGCAGGGCCTGCGCGGCAACCAGTAC
 DzCYP72A5    (95) GGAGGCTGGATCGGGAGCTCCGGCAGCAGGGCCTGCGCGGCAACCAGTAC
DzCYP72A10    (95) GGAGGCTGGATCGGGAGCTCCGGCGGCAGGGCCTGCGCGGCAACCAGTAC
 DzCYP72A3    (89) GGAGGCTGGATCGGGAGCTCCGGCGGCAGGGCCTGCGCGGCAACCAGTAC
 DzCYP72A6    (89) GGAGGCTGGACCGGGAGCTCCGGCGGCAGGGCCTGCGCGGCAACCAGTAC
DzCYP72A11    (95) TGAGGCTGGACCGGGAGCTCCGGCGCCAGGGCCTGCGCGGCAACCAGTAC
 DzCYP72A4    (95) TGAGGCTGGACCGGGAGCTCCGGCGCCAGGGCCTGCGCGGCAACCAGTAC
 DzCYP72A7     (2) TGAGGCTGGACCGGGAGCTCCGGCGCCAGGGCCTGCGCGGCAACCAGTAC
DzCYP72A12    (95) GGAGGCTGGACCGGGAGCTCCGACGCCAGGGCCTGCGCGGCAACCAGTAC
DzCYP72A13    (95) GGAGGCTGGACCGGGATCTCCGGCGCCAGGGCCTGCGCGGCAACCAGTAC
                   151                                              200
 DzCYP72A1   (145) CGACTCTTGCACGGCGATCTCAAGGAAAACGCCCGGCTCTCCGAGAAGGC
 DzCYP72A8   (145) CGACTCTTGCACGGCGATCTCAAGGAAAACGCCCGGCTCTCCGAGGAGGC
 DzCYP72A2   (151) CGAGTCTTGCACGGCGATCTCAAGGAAAACGCCCGGCTATCCGAGGAGGC
 DzCYP72A9   (151) CGAGTCTTGCACGGCGATCTCAAGGAAAACGCCCGGCTATCCGAGGAGGC
 DzCYP72A5   (145) CGAGTCTTGCACGGCGATCTCAAGGAAAACACCCGGCTATCCGAGGAGGC
DzCYP72A10   (145) CGACTCTTGCACGGCGATCTCAAGGAAAACGCCCAGCTCTCCGAGGAGGC
 DzCYP72A3   (139) CGACTCTTGCACGGCGATCTCAAGGAAAACGCCCAGCTCTCCGATGAAGC
 DzCYP72A6   (139) CGAGTATTGCACGGCGATCTCAAGGAAAACGCCCAGCTCTCCGAGGAGGC
DzCYP72A11   (145) CGAGTCTTCCATGGCGATCTCAAGGAAAACGCCCGGCTCTCGAAGGAGGC
 DzCYP72A4   (145) CGAGTCTTCCATGGCGATCTCAAGGAAAACGCCCGGCTCTCGAAGGAGGC
 DzCYP72A7    (52) CGAGTCTTCCATGGCGATCTCAAGGAAAACGCCCGGCTCTCGAAGGAGGC
DzCYP72A12   (145) CGAGTCTTGCACGGCGATCTCAAGGAAAACGTCCGGCTCTCGAAGGAGGC
DzCYP72A13   (145) CGATTATTGCACGGCGATCTCAAGGAAAACGCCCGGCTCTCCAAGGAGGC
                   201                                              250
 DzCYP72A1   (195) TAAATCCCGGCCTCTGCCTCTTCACTGCCATGACATCGCCCCCCGCGTTC
 DzCYP72A8   (195) TAAATCCCGGCCTCTGCCTCTTCACTGCCATGACATCGCCCCCCGCGTTC
 DzCYP72A2   (201) TAAATCCCGGCCTCTGCCTCTTCACTGCCATGACATCGCCCCCCGCGTTG
 DzCYP72A9   (201) TAAATCCCGGCCTCTGCCTCTTCACTGCCATGACATCGCCCCCCGCGTTG
 DzCYP72A5   (195) TAAATCCCGGCCTCTGCCTCTTCACTGCCATGACATCGCCCCCCGCGTTG
DzCYP72A10   (195) TAAATCCCGGCCTCTGCCTCTTCACTACCATGACATCGCCCCCCGCGTTC
 DzCYP72A3   (189) TAAATCCCGGCCTCTGCCTCTTCACTGCCATGACATCGCCCCCCGCGTTC
 DzCYP72A6   (189) TAAATCCCGGCCTCTGCCTCTTCACTACCATGACATCGCCCCCCGCGTTC
DzCYP72A11   (195) TGAATCCCGGCCTCTGCCTCTTCACTGCCATGACATCGCCCCCCGCGTTC
 DzCYP72A4   (195) TGAATCCCGGCCTCTGCCTCTTCACTGCCATGACATCGCCCCCCGCGTTC
 DzCYP72A7   (102) TGAATCCCGGCCTCTGCCTCTTCACTGCCATGACATCGCCCCCCGCGTTC
DzCYP72A12   (195) TAAATCCCGGCCTCTGCCTCTTCACTGCCATGACATCGCCCCCCGCGTTC
DzCYP72A13   (195) TACATCCCGGCCTTTGCCTCTTCACTGCCATGACATCGCTCCCAGGGTTG
```

```
                    251                                                 300
 DzCYP72A1    (245) TCCCTGTCCTCCACAACGCCATCAAAGATCACGGTAAAATCTCAATAACT
 DzCYP72A8    (245) TCCCTGTCCTCCACAACGCCATCAAAGATCACGGTAAAATCTCAATAACT
 DzCYP72A2    (251) TCCCTGTCCTCCACAACGCCATCAAAGATCACGGTAAAATCTCAATAACT
 DzCYP72A9    (251) TCCCTGTCCTCCACAACGCCATCAAAGATCACGGTAAAATCTCAATAACT
 DzCYP72A5    (245) TCCCTGTCCTCCACAACGCCATCAAAGATCACGGTAAAATCTCAATAACT
DzCYP72A10    (245) TCCCTCTCTTCCACAACGCCATCAAAGATCACGGTAAAATCTCAATAACT
 DzCYP72A3    (239) TCCCTCTCTTCCACAACGCCATCAAAGATCACGGTAAAATCTCAATAACT
 DzCYP72A6    (239) TCCCTCTCTTCCACAACGCCATCAAAGATCACG-----------------
DzCYP72A11    (245) TCCCTCTCTTCCACAACGCCATCAAAGATCATGGTAAAATCTCAATAACT
 DzCYP72A4    (245) TCCCTCTCTTCCACAATGCCATCAAAGATCACGGTAAAATCTCAATAACT
 DzCYP72A7    (152) TCCCTCTCTTCCACAACGCCATCAAAGATCACGGTAAAATCTCAATAACT
DzCYP72A12    (245) TCCCTCTCTTCCACAACGCCATCAAAGATCACGGTAAAATCTCAATAACT
DzCYP72A13    (245) CCCCTCTCATCCACAACGCCATCAAAGATCACGGTAAAATATCAATAACT
                    301                                                 350
 DzCYP72A1    (295) TGGTTTGGGCCTTACCCAAGAGTGACCTTAATGGAGCCAGAGCTAGTCAA
 DzCYP72A8    (295) TGGTTTGGGCCTTACCCAAGAGTGACCTTAATGGAGCCAGAGCTAGTCAA
 DzCYP72A2    (301) TGGTTTGGGCCTTACCCAAGAGTGACCTTAATGGAGCCAGAGCTAGTGAA
 DzCYP72A9    (301) TGGTTTGGGCCTTACCCAAGAGTGACCTTAATGGAGCCAGAGCTAGTGAA
 DzCYP72A5    (295) TGGTTTGGGCCTTACCCAAGAGTGACCTTAATGGAGCCAGAGCTAGTGAA
DzCYP72A10    (295) TGGCTTGGTCCTTACCCAAGAGTGATCTTAACAGAGCCAGAGCTAGTGAA
 DzCYP72A3    (289) TGGCTTGGTCCTTACCCAAGAGTGATCTTAGCAGAGCCAGAGCTAGTGAA
 DzCYP72A6    (272) ----------------------------------AGCCAGAGCTAGTGAA
DzCYP72A11    (295) TGGCTTGGCCCTTATCCAAGAGTGACCTTAACGGAACCAGAACTAGTTAT
 DzCYP72A4    (295) TGGCTTGGCCCTTGTCCAAGAGTGACCTTAACGGAACCAGAACTAGTAAA
 DzCYP72A7    (202) TGGCTTGGCCCTTGTCCAAGAGTGACCTTAACGGAACCAGAACTAGTAAA
DzCYP72A12    (295) TGGCTTGGCCCTTATCCAAGAGTGACCTTAACAGAGCCAGAGCTAGTGAA
DzCYP72A13    (295) TGGCTTGGACCTTATCCAAGAGTGTCCTTGATGGACCCAGATTTAGTGAA
                    351                                                 400
 DzCYP72A1    (345) AGAAGTGTTGTCAAACAAATTTGGACATTTTGCTAAAATAAGAGCAACTC
 DzCYP72A8    (345) AGAAGTGTTGTCAAACAAATTTGGACATTTTGCTAAAATAAGAGCAACTC
 DzCYP72A2    (351) AGAAGTGTTGTCAAACAAGTTTGGACATTTTGCTAAAGTAAGACCAAATC
 DzCYP72A9    (351) AGAAGTGTTGTCAAACAAGTTTGGACATTTTGCTAAAGTAAGACCAAATC
 DzCYP72A5    (345) AGAAGTGTTGTCAAACAAGTTTGGACATTTTGCTAAAGTAAGACCAAATC
DzCYP72A10    (345) AGAAGTACTCTCAAACAAGTTTGGACATTTTGTTAAACCAAGTACAACTC
 DzCYP72A3    (339) AGAAGTACTCTCAAACAAGTTTGGACATTTTGTTAAACCAAGTACAACTC
 DzCYP72A6    (288) AGAAGTACTCTCAAACAAGTTTGGACATTTTGTTAAACCAAGTACAACTC
DzCYP72A11    (345) AGAAGTGTT---AAACAAGTTTGGACATTTTGTTAAACCAAGTACAACTC
 DzCYP72A4    (345) AGAAGTGTT---AAACAAGTTTGGACATTTTGTTAAACCAAGTACAACTC
 DzCYP72A7    (252) AGAAGTGTT---AAACAAGTTTGGACATTTTGTTAAACCAAGTACAACTC
DzCYP72A12    (345) AGAAGTGCTGTCAAACAAGTTTGGACATTTTGTTAAACCAACTACAACTC
DzCYP72A13    (345) AGAAGTACTGTCAAACAAGTTTGGACATTTTGTTAAACCAAGAATAACTC
                    401                                                 450
 DzCYP72A1    (395) CCCTTGCCAATTTTTTGGTCCAAGGACTTGTGACTTATGAAGGTGAAAAG
 DzCYP72A8    (395) CCCTTGCCAATTTTTTGGTCCAAGGACTTGTGACTTATGAAGGTGAAAAG
 DzCYP72A2    (401) CCCTTACCAAATTATTGGTCCAAGGACTTGTAGTTTATGAAGGTGAAAAG
 DzCYP72A9    (401) CCCTTACCAAATTATTGGTCCAAGGACTTGTAGTTTATGAAGGTGAAAAG
 DzCYP72A5    (395) CCCTTACCAAATTATTGGTCCAAGGACTTGTAGTTTATGAAGGTGAAAAG
DzCYP72A10    (395) CCCTTGCCAAATTTTTGGTCCAAGGGCTTGCGTCTTATGACGGTGAAAAG
 DzCYP72A3    (389) CCCTTGCCAAATTTTTGGTCCAAGGGCTTGCGTCTTATGACGGTGAAAAG
 DzCYP72A6    (338) CCCTTGCCAAATTTTTGGTCCAAGGGCTTGCGTCTTATGACGGTGAAAAG
DzCYP72A11    (392) CTTTTGCCAAATTTTTGGTCCAAGGGCTTGTGTCTTATGAAGGTGAAAAG
 DzCYP72A4    (392) CTTTTGCCAAATTTTTGGTCCAAGGGCTTGTGTATTATGAAGGAGAACAC
 DzCYP72A7    (299) CTTTTGCCAAATTTTTGGTCCAAGGGCTTGTGTCTTATGAAGGTGAAAAG
DzCYP72A12    (395) CCTTAGCCAAATTTTTGGTCCAAGGGCTTGTGTCTTATGAAGGTGAAAAG
DzCYP72A13    (395) CTATTGCCAAATTTTTGGTCCAAGGACTTGTAGCTTATGAAGGTGAAAAG
                    451                                                 500
```

```
DzCYP72A1    (445) TGGGCCAAACATAGAAGGATCATCAACCCTGCATTCCATCTTGAGAAACT
DzCYP72A8    (445) TGGGCCAAACATAGAAGGATCATCAACCCTGCATTCCATCTTGAGAAACT
DzCYP72A2    (451) TGGGCCAAACACAGAAGGATCATCAACCCTGCATTCCATCTTGAGAAACT
DzCYP72A9    (451) TGGGCCAAACACAGAAGGATCATCAACCCTGCATTCCATCTTGAGAAACT
DzCYP72A5    (445) TGGGCCAAACACAGAAGGATCATCAACCCTGCATTCCATCTTGAGAAACT
DzCYP72A10   (445) TGGGTCAAACACAGGAGGATCATCAACCCTGCATTCCATTTTGAGAAACT
DzCYP72A3    (439) TGGGTCAAACACAGAAGGATCATCAACCCTGCATTCCATTTTGAGAAACT
DzCYP72A6    (388) TGGGTCAAACACAGAAGGATCATCAACCCTGCATTCCATTTTGAGAAACT
DzCYP72A11   (442) TGGGCCAAACATAGAAGGATCCTCAACCCTGCATTCCATCTTGAGAAACT
DzCYP72A4    (442) TGGGCCAAACATAGAAGGATACTCAACCCTGCATTCCATCTTGAGAAACT
DzCYP72A7    (349) TGGGCCAAACATAGAAGGATCCTCAACCCTGCATTCCATCTAGAGAAACT
DzCYP72A12   (445) TGGGCCAAACACAGAAGGATCATCAATCCTGCATTCCATCTTGAGAAACT
DzCYP72A13   (445) TGGGCCAAACACAGAAGGATAATTAACCCCGCATTCCATCTTGAGAAACT
                   501                                              550
DzCYP72A1    (495) AAAGCTAATGCTTCCAGCATTCTCTACATCTTGTGGTGAACTGATTAGAA
DzCYP72A8    (495) AAAGCTAATGCTTCCAGCATTCTCTACATCTTGTGGTGAACTGATTAGAA
DzCYP72A2    (501) AAAGCTAATGCTTCCAGCATTCTCTACATCTTGTGGTGAACTGATTAGAA
DzCYP72A9    (501) AAAGCTAATGCTTCCAGCATTCTCTACATCTTGTGGTGAACTGATTAGAA
DzCYP72A5    (495) AAAGCTAATGCTTCCAGCATTCTCTACATCTTGTGGTGAACTGATTAGAA
DzCYP72A10   (495) AAAGCAAATGCTGCCAGCATTCTCTACGTCTTGTGGTGAACTTATTAGAA
DzCYP72A3    (489) AAAGCAAATGCTGCCAGCATTCTCTACGTCTTGTGGTGAACTTATTAGAA
DzCYP72A6    (438) AAAGCAAATGCTGCCAGCATTCTCTACGTCTTGTGGTGAACTTATTAGAA
DzCYP72A11   (492) AAAGCTAATGTTGCCAGCATTCTCTACATCTTGTAGTGAATTGATTAGAA
DzCYP72A4    (492) AAAGCTAATGTTGCCAGCATTCTCTACATCTTGTAGTGAATTGATTAGAA
DzCYP72A7    (399) AAATCTAATGTTGCCAGCATTCTCTACATCTTGTAGTGAATTGATTAGAA
DzCYP72A12   (495) AAAGCTAATGCTGCCAGCATTCTCTACATCTTCTGGTGAACTGATTAGAA
DzCYP72A13   (495) AAAGCTAATGCTGCCAGCATTCTCTACATCTTCTGGTGAACTGATTAGAA
                   551                                              600
DzCYP72A1    (545) GATGGGAGAAGATGATCCCTGATGAAGGCTCCCAAGAACTAAAATGTCTT
DzCYP72A8    (545) GATGGGAGAAGATGATCCCTGATGAAGGCTCCCAAGAACTAAA-TGTCTT
DzCYP72A2    (551) GATGGGAGAAGATGATCCCTGATGAAGGCTCCCATGAACTAAA-TGTCTT
DzCYP72A9    (551) GATGGGAGAAGATGATCCCTGATGAAGGCTCCCATGAACTAAA-TGTCTT
DzCYP72A5    (545) GATGGGAGAAGATGATCCCTGATGAAGGCTCCCATGAACTAAA-TGTCTT
DzCYP72A10   (545) GATGGAACAAGATGATCCCTGATGAAGGCTCCCAAGAACTAAA-TGTCTT
DzCYP72A3    (539) GATGGAACAAGATGATCCCTGATGAAGGCTCCCAAGAACTAAA-TGTCTT
DzCYP72A6    (488) GATGGAACAAGATGATCCCTGATGAAGGCTCCCAAGAACTAAA-TGTCTT
DzCYP72A11   (542) GATGGGAGGAGAAGATTCCTGATGAAGGCTCCCAAGAACTAAA-TGTCTT
DzCYP72A4    (542) GATGGGAGAAGATGATTCCTGATGAAGGCTCCCAAGAACTAAA-TGTCTT
DzCYP72A7    (449) GATGGGAGAAGATGATTCCTGATGAAGGCTCCCAAGAACTAAA-TGTCTT
DzCYP72A12   (545) GATGGGAGAAGATGATTCCTAATGAAGGCTCCCAAGAACTAAA-TGTCTT
DzCYP72A13   (545) GATGGGAGAAGATGATCCCTGATGAAGGCTCCCAAGAACTAAA-TGTCTC
                   601                                              650
DzCYP72A1    (595) TCCAGAGCTCCAAGACCTCACAAAAGATGTCATCTCCAGGACTGCATTCG
DzCYP72A8    (594) TCCAGAGCTCCAAGACCTCACAAAAGATGTCATCTCCAGGACTGCATTCG
DzCYP72A2    (600) TCCAGAGCTCCAAGACCTCACAAAAGATGTCATCTCCAGGACTGCATTCG
DzCYP72A9    (600) TCCAGAGCTCCAAGACCTCACAAAAGATGTCATCTCCAGGACTGCATTCG
DzCYP72A5    (594) TCCAGAGCTCCAAGACCTCACAAA-GATGTCATCTCCAGGACTGCATTCG
DzCYP72A10   (594) TCCAGAGCTCCAAGGCCTCACAAAAGATATCATCTCCAGAACCGCATTCG
DzCYP72A3    (588) TCCAGAGCTCCAAGGCCTCACAAAAGATATCATCTCCAGAACCGCATTCG
DzCYP72A6    (537) TCCAGAGCTCCAAGGCCTCACAAAAGATATCATCTCCAGAACCGCATTCG
DzCYP72A11   (591) TCCAGAGCTCCAAGGCCTCACAAAAGATGTCATCTCTAGGACTGCATTCA
DzCYP72A4    (591) TCCAGAGCTCCAAGGCCTCACAAAAGATGTCATCTCTAGGACTGCATTCA
DzCYP72A7    (498) TCCAGAGCTCCAAGGCCTCACAAAAGATGTCATCTCTAGGACTGCATTCA
DzCYP72A12   (594) TCCAGAGCTCCAAGACCTCACAAAAGATGTCATCTCCAGGACTGCATTCG
DzCYP72A13   (594) GCCAGAGATCCAAAACCTCACAGGAGATGTCATCTCCAGGACTGCATTCG
                   651                                              700
DzCYP72A1    (645) -TAGCAGCTATGAAGAAGGAAG----AAGAATATTTGAACTCCTAGCAGA
```

```
DzCYP72A8    (644) GTAGCAGCTATGAAGAAGGAAG----AAGAATATTTGAACTCCTAGCAGA
DzCYP72A2    (650) GTAGCAGCTATGAAGAAGGAAG----AAGAATATTTGAACTCCTAGCAGA
DzCYP72A9    (650) GTAGCAGCTATGAAGAAGGAAG----AAGAATATTTGAACTCCTAGCAGA
DzCYP72A5    (643) GTAGCAGCTATGAAGAAGGAAGGAAGAAGAATATTTGAACTACTAGCAGA
DzCYP72A10   (644) GTAGCAGCTATGAAGAAGGGAG----AAGAATATTTGAACTCCTAACTGA
DzCYP72A3    (638) GTAGCAGCTATGAAGAAGGGAG----AAGAATATTTGAACTCCTAACTGA
DzCYP72A6    (587) GTAGCAGCTATGAAGAAGGGAG----AAGAATATTTGAACTCCTAACTGA
DzCYP72A11   (641) GTAGCAGCTATGAAGAAGGGAG----AAGAATATTTGAACTCCTAAAAGA
DzCYP72A4    (641) GTAGCAGCTATGAAGAAGGGAG----AAGAATATTTGAACTCCTAAAAGA
DzCYP72A7    (548) GTAGCAGCTATGAAGAAGGGAG----AAGAATATTTGAACTCCTAAAAGA
DzCYP72A12   (644) GTAGCAGTTACGAAGACGGAAG----AAGAATATTTGAACTCCTAACAGA
DzCYP72A13   (644) GTAGCAGCTATGAAGAAGGGAG----AAGAATATTTGAACTCCTAACAGA
                   701                                             750
DzCYP72A1    (690) GCAAATTCAGCTTCTTATCCCAGCTTTCCAGACTATATACATCCCTGGTT
DzCYP72A8    (690) GCAAATTCAGCTTCTTATCCCAGCTTTCCAGACTATATACATCCCTGGTT
DzCYP72A2    (696) GCAAATTCAGCTTCTTATCCCAGCTTTCCAGACTATATACATCCCTGGTT
DzCYP72A9    (696) GCAAATTCAGCTTCTTATCCCAGCTTTCCAGACTATATACATCCCTGGTT
DzCYP72A5    (693) GCAAATTCAGCTTCTTATCCCAGCTTTCCAGACTTTATACATCCCTGGTT
DzCYP72A10   (690) ACAAATTAAGCTTACTATTCCAGCTTTCAAGACTGTATACATCCCTGGTT
DzCYP72A3    (684) ACAAATTAAGCTTACTATTCCAGCTTTCAAGACTGTATACATCCCTGGTT
DzCYP72A6    (633) ACAAATTAAGCTTACTATTCCAGCTTTCAAGACTGTATACATCCCTGGTT
DzCYP72A11   (687) GCAAATTCAGCTTTATATTCAAGTTTACAAGACTGTATACATCCCTGGTT
DzCYP72A4    (687) GCAAATTCAGCTTTATATTCAAGTTTACAAGACTGTATACATCCCTGGTT
DzCYP72A7    (594) GCAAATTCAGCTTTATATTCAAGTTTACAAGACTGTATACATCCCTGGTT
DzCYP72A12   (690) GCAAATTCAGCTTCTTATTCCAGCTTTCCAGACTGTATACATTCCTGGTT
DzCYP72A13   (690) GCAAATTCATCTTACTATTCCAGCTTTGCAGACTGTATACATCCCTGGTT
                   751                                             800
DzCYP72A1    (740) ATCGATTTCTGCCCACACCAATGAACAAAAGAAGAAGCCAAGTGTACAAT
DzCYP72A8    (740) ATCGATTTCTGCCCACACCAATGAACAAAAGAAGAAGCCAAGTGTACAAT
DzCYP72A2    (746) ATCGATTTCTGCCCACACCAATGAACAAAAGAAGAAGCCAAGTGTACAAT
DzCYP72A9    (746) ATCGATTTCTGCCCACACCAATGAACAAAAGAAGAAGCCAAGTGTACAAT
DzCYP72A5    (743) ATCGATTTCTGCCCACACCAATGAACAAAAGAAGAAGCCAAGTGTACAAT
DzCYP72A10   (740) ATCAATTTCTGCCCACACCAATGAACAAAAGAAGAAGCCAAGTGTACAAT
DzCYP72A3    (734) ATCGATTTCTGCCCACACCAATGAACAAAAGAAGAAGCCAAGTGTACAAT
DzCYP72A6    (683) ATCGATTTCTGCCCACACCAATGAACAAAAGAAGAAGCCAAGTGTACAAT
DzCYP72A11   (737) ATCGATTTCTGCCCACACCAATGAACAAAAGAAGAAGCCAAGTATATAAT
DzCYP72A4    (737) ATCGATTTCTGCCCACACCAATGAACAAAAGAAGAAGCCAAGTATACAAT
DzCYP72A7    (644) ATCGATTTCTGCCCACACCAATGAACAAAAGAAGAAGCCAAGTATACAAT
DzCYP72A12   (740) TTCGATTTCTGCCCACACCAATGAACAAAAGAAGCAGCCAAGTGTACAAT
DzCYP72A13   (740) ATCGGTTTCTGCCCACACCAATGAACAACAGAAGAAGCCAAGTTTACAAT
                   801                                             850
DzCYP72A1    (790) GAGATGAAAAGAATTCTTAAAGGCATGATAG-AGAAGAGAGAGAAGGCCA
DzCYP72A8    (790) GAGATGAAAAGAATTCTTAAAGGCATGATCG-AGAAGAGAGAGAAGGCCA
DzCYP72A2    (796) GAGATGAAAAGAATTCTTAAAGGCATGATAG-AGAAGAGAGAGAAGGCCA
DzCYP72A9    (796) GAGATGAAAAGAATTCTTAAAGGCATGATAG-AGAAGAGAGAGAAGGCCA
DzCYP72A5    (793) GAGATGAAAAGAATTCTTAAAGGCATGATCG-AGAAGAGAGAGAAGGCCA
DzCYP72A10   (790) GAGATGAAAAGAATTCTTAAAGGCATGATTG-AGAAGAGAGAGAAGGCCA
DzCYP72A3    (784) GAGATGAAAAGAATTCTTAAAGGCATGATTG-AGAAGAGAGAGAAGGCCA
DzCYP72A6    (733) GAGATGAAAAGAATTCTTAAAGGCATGATTG-AGAAGAGAGAGAAGGCCA
DzCYP72A11   (787) GAGATGAAAAGAATTCTTAAAGGCATGATTG-AGAAGAGAGAAAAGGCTA
DzCYP72A4    (787) GAGATGAAAAGAATTCTTAAAGGCATGATTGGAGAAGAGAGAAAAGGCCA
DzCYP72A7    (694) GAGATGAAAAGAATTCTTAAAGGCATGATTG-AGAAGAGAGAAAAGGCCA
DzCYP72A12   (790) GAGATGAAAAGAATTCTTATAGGCATGATTG-AGAAGAGAGAGAAGGCCA
DzCYP72A13   (790) GAGATGAAAAGAATTCTTAAAGGCATGATTG-AGAAGAGAGAGAAGGCCA
                   851                                             900
DzCYP72A1    (839) TAAGAATGGGGGACAGTAGCAAGAATGACCTTCTGGGTTTGTTACTAGAC
DzCYP72A8    (839) TAAGAATGGGGGAAGGTAGCAAGAATGACCTTCTGGGTTTGTTACTAGAC
```

```
DzCYP72A2    (845)  TAAGAATGGGGGACAGTAGCAAGAATGACCTTCTGGGTTTGTTACTAGAC
DzCYP72A9    (845)  TAAGAATGGGGGACAGTAGCAAGAATGACCTTCTGGGTTTGTTACTAGAC
DzCYP72A5    (842)  TAAGAATGGGGGAAGGTAGCAAGAATGACCTTCTGGGTTTGTTACTAGAC
DzCYP72A10   (839)  TAAGAATGGGGGAAAGTAGCAAGAATGACCTTCTGGGTTTGCTACTAGAC
DzCYP72A3    (833)  TAAGAATGGGGGAAAGTAGCAAGAATGACCTTCTGGGTTTGCTACTAGAC
DzCYP72A6    (782)  TAAGAATGGGGGAAAGTAGCAAGAATGACCTTCTGGGTTTGCTACTAGAC
DzCYP72A11   (836)  TAAGAATGGGGGAAAGTTGTAAGGATGACCTTCTGGGTTTGTTACTAGAC
DzCYP72A4    (837)  TAAGAATGGGG-AAAGTTGTAAGGATGACCTTCTGGGTTTGTTACTAGAC
DzCYP72A7    (743)  TAAGAATGGGGGAAAGTTGTAAGGATGACCTTCTGGGTTTGTTACTAGAC
DzCYP72A12   (839)  TAAGAATGGGGGAAAGTAACAAGAATGACCTTCTGGGTTTGTTACTAGAC
DzCYP72A13   (839)  TAAGAATGGGGGAAAGTAGCAAGAATGACCTTCTGGGTTTGTTACTAGAC
                 901                                                950
DzCYP72A1    (889)  TCCAATATGAAGGAGGGTGAAGAGCATGGGAAGTCCCAAAATAAAGGGAT
DzCYP72A8    (889)  TCCAATATCAAAGAGAGTGAAGAGCATGGGAAGTCCCAAAACAAAGTGAT
DzCYP72A2    (895)  TCCAATATGAAGGAGGGTGAAGAGCATGGGAAGTCCCAAAACAAAGGGAT
DzCYP72A9    (895)  TCCAATATGAAGGAGGGTGAAGAGCATGGGAAGTCCCAAAACAAAGGGAT
DzCYP72A5    (892)  TCCAATATCAAAGAGAGTGAAGAGCATGGGAAGTCCCAAAACAAAGTGAT
DzCYP72A10   (889)  TCCAATATGCAGGAGGGTGAAGAGCATGGGAAGTCCCAAGACAAAGGGAT
DzCYP72A3    (883)  TCCAATATGCAGGAGGGTGAAGAGCATGGGAAGTCCCAAGACAAAGGGAT
DzCYP72A6    (832)  TCCAATATGCAGGAGGGTGAAGAGCATGTGAAGTCCCAAGACAAAGGGAT
DzCYP72A11   (886)  TCCAATATGAAGGAGGGTGAAGAGCATGGCAAGACCCAAAACAAAGGGAT
DzCYP72A4    (886)  TCCAATATGAAGGAGGGTGAAGAGCATGGCAAGTCCCAAAACAAAGGGAT
DzCYP72A7    (793)  TCCAATATGAAGGAGGGTGAAGAGCATGGCAAGTCCCAAAACAAAGGGAT
DzCYP72A12   (889)  TCCAATATGAAGGAGGGTGAAGAGCATAGGAAGTCCCAAAACAAAGGGAT
DzCYP72A13   (889)  TCCAATATGAAGGAGGGTGAAGAGCAGGAGAAGTCCCAAAACAAAGTGAT
                 951                                                1000
DzCYP72A1    (939)  GAGCACTGAGGATGTGATTGAAGAGTGCAAGCTGTTCTACTTTGCAGGGC
DzCYP72A8    (939)  GAGCACTGAGGATGTGATTGAAGAGTGCAAGCTATTCTACTTTGCAGGGC
DzCYP72A2    (945)  GAGCACTGAGGATGTGATTGAAGAGTGCAAGCTGTTCTACTTTGCAGGGC
DzCYP72A9    (945)  GAGCACTGAGGATGTGATTGAAGAGTGCAAGCTGTTCTACTTTGCAGGGC
DzCYP72A5    (942)  GAGCACTGAGGATGTGATTGAAGAGTGCAAGCTATTCTACTTTGCAGGGC
DzCYP72A10   (939)  GAGCACTGAGGATGTGATTGAAGAGTGCAAGCTGTTTTACATTGCAGGGC
DzCYP72A3    (933)  GAGCACTGAGGATGTGATTGAAGAGTGCAAGCTGTTTTACATTGCAGGGC
DzCYP72A6    (882)  GAGCACTGAGGATGTGATTGAAGAGTGCAAGCTGTTTTACATTGCAGGGC
DzCYP72A11   (936)  GAGCACTGAGGAAGTGATTGAAGAGTGCAAGCTGTTCTACTTTGCAGGGC
DzCYP72A4    (936)  GAGCACTGAGGAAGTGATTGAAGAGTGCAAGCTGTTCTACTTTGCAGGGC
DzCYP72A7    (843)  GAGCACTGAGGAAGTGATTGAAGAGTGCAAGCTGTTCTACTTTGCAGGGC
DzCYP72A12   (939)  GAGTACTGAGGATGTGATTGAAGAGTGCAAGCTGTTCTACTTTGCAGGGC
DzCYP72A13   (939)  GAGCACTGAGGATGTGATTGAAGAGTGCAAGCTGTTCTACTTTGCAGGGC
                 1001                                               1050
DzCYP72A1    (989)  AAGAGACTACA---GCAGTTCTACTCACATGGACAATGATTTTATTGAGC
DzCYP72A8    (989)  AAGAGACTACA---TCACTTTTACTCACATGGACAATGATTTTATTGAGC
DzCYP72A2    (995)  AAGAGACTACA---GCAGTTCTACTCACATGGACAATGATTTTATTGAGC
DzCYP72A9    (995)  AAGAGACTACA---GCAGTTCTACTCACATGGACAATGATTTTATTGAGC
DzCYP72A5    (992)  AAGAGACTACA---TCACTTTTACTCACATGGACAATGATTTTATTGAGT
DzCYP72A10   (989)  AAGAGACTACA---TCAGCTCTACTCACTTGGACAATGATTTTATTGAGC
DzCYP72A3    (983)  AAGAGACTACA---TCAGCTCTACTCACTTGGACAATGATTTTATTGAGC
DzCYP72A6    (932)  AAGAGACTACA---TCAGCTCTACTCACTTGGACAATGATTTTATTGAGC
DzCYP72A11   (986)  AAGAGACTACC---TCACTTTTACTCACATGGACAATGATTTTATTGAGC
DzCYP72A4    (986)  AAGAGACTACCCCCTCACTTTTACTCACATGGACAATGATTTTATTGAGC
DzCYP72A7    (893)  AAGAGACTACC---TCACTTTTACTCACATGGACAATGATTTTATTGAGC
DzCYP72A12   (989)  AAGAGACTACA---TCAGTTCTACTCACATGGACAATGATTTTATTGAGC
DzCYP72A13   (989)  AAGAGACTACA---TCAGTTCTACTCACATGGACAATGATTTTATTGAGC
                 1051                                               1100
DzCYP72A1   (1036)  ATGTATCCAAACTGGCAGGCTAAGGCAAGAGAAGAGGTACTTCAAGTCTT
DzCYP72A8   (1036)  ATGTATCCAAACTGGCAGGGTAAGGCAAGAGAAGAGGTACTTCAAGTCTT
DzCYP72A2   (1042)  ATGTATCCAAACTGGCAGGGTAAGGCAAGAGAAGAGGTACTTCAAGTCTT
```

```
 DzCYP72A9  (1042) ATGTATCCAAACTGGCAGGCTAAGGCAAGAGAAGAGGTACTTCAAGTCTT
 DzCYP72A5  (1039) ATGTATCCGAACTGGCAGGCTAAGGCAAGAGAAGAGGTTCTTCAAGTCTT
DzCYP72A10  (1036) ATGTATCCGAACTGGCAGGCTAATGCAAGAGAAGAGGTTCTTCAAGTCTT
 DzCYP72A3  (1030) ATGTATCCGAACTGGCAGGCTAATGCAAGAGAAGAGGTTCTTCAAGTCTT
 DzCYP72A6   (979) ATGTATCCGAACTGGCAGGCTAATGCAAGAGAAGAGGTTCTTCAAGTCTT
DzCYP72A11  (1033) ATGTATCCGAACTGGCAGGCTAAGGCAAGAGAAGAGGTTCTTCAAGTCTT
 DzCYP72A4  (1036) ATGTATCCGAACTGGCAGACTAAGGCAAGAGAAGAGGTTCTTAAAGTCTT
 DzCYP72A7   (940) ATGTATCCGAACTGGCAGACTAAGGCAAGAGAAGAGGTTCTTAAAGTCTT
DzCYP72A12  (1036) ATGTATCCAAATTGGCAGGCTAAGGCAAGAGAAGAGGTTCTTCAAGTCTT
DzCYP72A13  (1036) ATGTATCCTAACTGGCAGGCCAAGGCAAGAGAAGAGGTTCTTCAAGTCTT
                   1101                                            1150
 DzCYP72A1  (1086) TGGAAAGAACACACCAGACATGGAGGGATTGAGCCACTTGAAGATTGTGA
 DzCYP72A8  (1086) TGGAAAGAGCACACCAGATATGGAGGGATTGAACCACTTGAAGATTGTGA
 DzCYP72A2  (1092) TGGAAAGAGCACACCAGATATGGAGGGATTGAACCACTTGAAGATTGTGA
 DzCYP72A9  (1092) TGGAAAGAGCACACCAGATATGGAGGGATTGAACCACTTGAAGATTGTGA
 DzCYP72A5  (1089) TGGAAAGAACACACCAGACATGGAGGGATTGAGCCACTTGAAGATTGTGA
DzCYP72A10  (1086) TGGAAAGAGCACACCAGACATAGAGGGATTGAGCCACTTGAAGATTGTGA
 DzCYP72A3  (1080) TGGAAAGAGCACACCAGACATAGAGGGATTGAGTCACTTGAAGATTGTGA
 DzCYP72A6  (1029) TGGAAAGAGCACACCAGACATAGAGGGATTGAGTCACTTGAAGATTGTGA
DzCYP72A11  (1083) TGGAAAGAACACACCAGACATGGAGGGATTGAGCCACTTGAAGATTGTGA
 DzCYP72A4  (1086) TGGAAAGAACACACCAGACATGGAGGGATTGAGCCACTTGAAGATTGTGA
 DzCYP72A7   (990) TGGAAAGAACACACCAGACATGGAGGGATTGAGCCACTTGAAGATTGTGA
DzCYP72A12  (1086) TGGAAAGAGCGCACCAGATATGGAGGGATTGAGCCACTTGAAGATTGTGA
DzCYP72A13  (1086) TGGAAAGAGCACACCAGACATGGATGGATTGAGCCGCTTGAAGATTGTGA
                   1151                                            1200
 DzCYP72A1  (1136) CCATGATTCTATATGAAGTTCTGAGGTTATATCCACCATTTGTTTTACTA
 DzCYP72A8  (1136) CCATGATTCTATATGAAGTTCTGAGGTTATATCCACCATTTGTTTTCCTA
 DzCYP72A2  (1142) CCATGATTCTATATGAAGTTCTGAGGTTATATCCACCATTTGTTTTCCTA
 DzCYP72A9  (1142) CCATGATTCTATATGAAGTTCTGAGGTTATATCCACCATTTGTTTTCCTA
 DzCYP72A5  (1139) CCATGATTCTATATGAAGTTCTGAGGTTATATCCACCATTTGTTTTACTA
DzCYP72A10  (1136) CCATGATTCTATATGAAGTTCTCAGGTTATATCCACCAGGGGTTTTTCTT
 DzCYP72A3  (1130) CCATGATTCTATATGAAGTTCTCAGGTTATATCCACCAGGGGTTTTTCTT
 DzCYP72A6  (1079) CCATGATTCTATATGAAGTTCTCAGGTTATATCCACCAGGGGTTTTTCTT
DzCYP72A11  (1133) CCATGATTCTATATGAAGTTCTTAGGTTATATCCACCAGTGGTTTTCCTA
 DzCYP72A4  (1136) CCATGATTCTATATGAAGTTCTTAGGTTATATCCACCAGTGGTTTTCATA
 DzCYP72A7  (1040) CCATGATTCTATATGAAGTTCTTAGGTTATATCCACCAGTGGTTTTCATA
DzCYP72A12  (1136) CCATGATTCTATATGAAGTTCTAAGGTTATATCCACCAGCGGTTTTCCTA
DzCYP72A13  (1136) CCATGATTCTGTATGAAGTTCTGAGGTTATATCCACCGGTGACTTTCCTA
                   1201                                            1250
 DzCYP72A1  (1186) AGAAGAAAAACCTACAAAGCAATGGAACTCGGTGGGATTATTTACCCTCC
 DzCYP72A8  (1186) ACAAGAAAAACCTACAAAGCAATGGAACTCGGTGGGATTACTTACCCTCA
 DzCYP72A2  (1192) ACAAGAAAAACCTACAAAGCAATGGAACTCGGTGGGATTACTTACCCTCA
 DzCYP72A9  (1192) ACAAGAAAAACCTACAAAGCAATGGAACTCGGTGGGATTACTTACCCTCA
 DzCYP72A5  (1189) AGAAGAAAAACCTACAAAGCAATGGAACTCGGTGGGATTACTTACCCTCC
DzCYP72A10  (1186) GATAGAAAAACCTACAAAGCAATGGAACTTGGTGGGATTACTTACCCTTC
 DzCYP72A3  (1180) GATAGAAAAACCTACAAAGCAATGGAACTTGGTGGGATTACTTACCCTTC
 DzCYP72A6  (1129) GATAGCAAAACCTCCAAAGCAATGGAACTTGGTGGGATTACTTACCCTTC
DzCYP72A11  (1183) ACAAGAAAAACCTACAAAGCAATGGAACTCGGTGGGATTACTTACCCTCC
 DzCYP72A4  (1186) ACAAGAAAAACCTACAAAGCAATGGAACTCGGTGGGATTACTTACCCTCC
 DzCYP72A7  (1090) ACAAGAAAAACCTACAAAGCAATGGAACTCGGTGGGATTACTTACCCTCC
DzCYP72A12  (1186) ACAAGAAAAACCTACAAAGCAATGGAACTCGGTGGGATTACTTACCCTCC
DzCYP72A13  (1186) ACCAGAAAAACATACAAAGCAATGGAACTTGGTGGGATCAATTACCCTCC
                   1251                                            1300
 DzCYP72A1  (1236) GGGAGTGATGCTCTCACTGCCTCTACTCTTCATTCACCATGACCCTGCTT
 DzCYP72A8  (1236) AGGAGTGATACTCTCACTGCCTCTACTCTTCATTCACCATGACCCTGCTT
 DzCYP72A2  (1242) AGGAGTGATACTCTCACTGCCTCTACTCTTCATTCACCATGACCCTGCTT
 DzCYP72A9  (1242) AGGAGTGATACTCTCACTGCCTCTACTCTTCATTCACCATGACCCTGCTT
```

```
 DzCYP72A5  (1239) GGGAGTGATACTCTCACTGCCTCTACTCTTCATTCACCATGACCCTGCTT
DzCYP72A10  (1236) AGGAGTGATACTCTCACTACCTCTACTCTTCATTCACAATGACCCTACTT
 DzCYP72A3  (1230) AGGAGTGATACTCTCACTACCTCTACTCTTCATTCACAATGACCCTACTT
 DzCYP72A6  (1179) AGGAGTGATACTCTCACTACCTCTACTCTTCATTCACAATGACCCTACTT
DzCYP72A11  (1233) AGGAGTGATATTCTCATTGCCTTTACTCTTCATTCACCATGACCCTACTT
 DzCYP72A4  (1236) AGGAGTGATATTCTCATTGCCTTTACTCTTCATTCACCATGACCCTACTT
 DzCYP72A7  (1140) AGGAGTGATATTCTCATTGCCTTTACTCTTCATTCACCATGACCCTACTT
DzCYP72A12  (1236) AGGAGTGATACTCTCACTGCCTCTACTCTTCATTCACCATGACCCTGTTT
DzCYP72A13  (1236) AGGAGTGTTACTCCAACTGCCTCTACTCTTCATTCACCATGACCCTGAAT
                   1301                                            1350
 DzCYP72A1  (1286) TTTGGGGAGAAGACGCCAAAGAGTTTAATCCAGAGAGGTTTTCCGAAGGG
 DzCYP72A8  (1286) TTTGGGGAGAAGACGCCAAAGAGTTTAATCCAGAGAGGTTTTCCGAAGGG
 DzCYP72A2  (1292) TTTGGGGAGAAGACGCCAAAGAGTTTAATCCAGAGAGGTTTTCCGAAGGG
 DzCYP72A9  (1292) TTTGGGGAGAAGACGCCAAAGAGTTTAATCCAGAGAGGTTTTCCGAAGGG
 DzCYP72A5  (1289) TTTGGGGAGAAGACGCCAAAGAGTTTAATCCAGAGAGGTTTTCCGAAGGG
DzCYP72A10  (1286) TCTGGGGAGAGGATGCCAAAGAGTTCAATCCAGAGAGGTTTTCCGAAGGG
 DzCYP72A3  (1280) TCTGGGGAGAGGATGCCAAAGAGTTCAATCCAGAGAGGTTTTCCGAAGGG
 DzCYP72A6  (1229) TCTGGGGAGAGGATGCCAAAGAGTTCAATCCAGAGAGGTTTTCCGAAGGG
DzCYP72A11  (1283) TTTGGGGAGAAGATGCCAAAGAGTTTAATCCAGAGAGGTTTTCTGAAGGG
 DzCYP72A4  (1286) TTTGGGGAGAAGATGCCAAAGAGTTTAATCCAGAGAGGTTTTCCGAAGGG
 DzCYP72A7  (1190) TTTGGGGAGAAGATGCCAAAGAGTTTAATCCAGAGAGGTTTTCCGAAGGG
DzCYP72A12  (1286) TCTGGGGAGAAGACGCCAAAGAGTTTAATCCAGAGAGGTTTTCCGAAGGG
DzCYP72A13  (1286) TCTGGGGAGAAGACGCCAAAGAGTTCAATCCCGAGAGGTTTTCTGAAGGG
                   1351                                            1400
 DzCYP72A1  (1336) ATATCAAAAGCATCCAAAGTTCCAGGTGCCTTCTTTCCTTTCGGTGGAGG
 DzCYP72A8  (1336) ATATCAAAAGCATCCAAAGTTCCGGGTGCCTTCTTTCCTTTCGGTGGAGG
 DzCYP72A2  (1342) ATATCGAAAGCATCCAAAGTTCCGGGTGCCTTCTTTCCTTTCGGTGGAGG
 DzCYP72A9  (1342) ATATCGAAAGCATCCAAAGTTCCGGGTGCCTTCTTTCCTTTCGGTGGAGG
 DzCYP72A5  (1339) ATATCGAAAGCATCCAAAGTTCCAGGTGCCTTCTTTCCTTTCGGTGGAGG
DzCYP72A10  (1336) ATATCGAAAGCATCCAAAGTTCCAGGGTCCTTCTTACCTTATGGTGTAGG
 DzCYP72A3  (1330) ATATCAAAAGCATCCAAAGTTCCAGGGGCCTTCTTTCCTTTTGGTGGAGG
 DzCYP72A6  (1279) ATATCGAAAGCATCCAAAGTTACAGGGGCCTTCTTTCCTTTTGGTGGAGG
DzCYP72A11  (1333) ATATCGAAAGCATCCAAAGTTTCGGCTGCCTTCTTTCCTTTCGGTGGAGG
 DzCYP72A4  (1336) ATATCGAAAGCATCCAAAGTTCCGGCTGCCTTCTTTCCTTTCGGTGGAGG
 DzCYP72A7  (1240) ATATCGAAAGCATCCAAAGTTCCGGCTGCCTTCTTTCCTTTCGGTGGAGG
DzCYP72A12  (1336) ATATCGAAAGCATCCAAAGTTCCAGGTGCCTTCTTTCCTTTCGGTGGAGG
DzCYP72A13  (1336) ATATTGAAAGCATCCAAAGTTCCGGGTGCCTTCTTTCCTTTCGGTGGAGG
                   1401                                            1450
 DzCYP72A1  (1386) TCCACGCATTTGCATTGGCCAAAGCTTTGCAATGATTGAAGCTAAGATAG
 DzCYP72A8  (1386) TCCGCGCATTTGCATTGGCCAAAGCTTTGCAATGATTGAAGCTAAGATAG
 DzCYP72A2  (1392) TCCGCGCATTTGCATTGGCCAAAGCTTTGCAATGATTGAAGCTAAGATAG
 DzCYP72A9  (1392) TCCGCGCATTTGCATTGGCCAAAGCTTTGCAATGATTGAAGCTAAGATAG
 DzCYP72A5  (1389) TCCACGCATTTGCATTGGCCAAAGCTTTGCAATGATTGAAGCTAAGATAG
DzCYP72A10  (1386) TCCGCGCATTTGCATTGGCCAAAACTTTGCACTGATGGAAGCTAAGATAG
 DzCYP72A3  (1380) TCCGCGCATTTGCATTGGCCAAAACTTTGCACTGATGGAAGCTAAGATAG
 DzCYP72A6  (1329) TCCGCGCATTTGCATTGGCCAAAACTTTGCACTGATGGAAGCTAAGATAG
DzCYP72A11  (1383) TCCACGCATTTGCATTGGTCAAAACTTTGCACTGATTCAAGCTAAGATAG
 DzCYP72A4  (1386) TCCACGCATTTGCATTGGTCAAAACTTTGCACTGATTGAAGCTAAGATAG
 DzCYP72A7  (1290) TCCACGCATTTGCATTGGTCAAAACTTTGCACTGATTGAAGCTAAGATAG
DzCYP72A12  (1386) TCCGCGCATTTGCATTGGCCAAAACTTTGCACTGATTGAAGCTAAGATAG
DzCYP72A13  (1386) TCCGCGCATTTGCATTGGCCAAAGCTTTGCACTGATTGAAGCTAAGATTG
                   1451                                            1500
 DzCYP72A1  (1436) GAATTTGCATGATTCTTCAGTGTTTCTCCTTTGAGCTTTCAC---CTTCC
 DzCYP72A8  (1436) GAATTTGCATGATGCTTCAGCGCTTCTCCTTTGAGCTTTCGC---CTTCC
 DzCYP72A2  (1442) GAATTTGCATGATTCTTCAGCGCTTCTCCTTTGAGCTTTAA---------
 DzCYP72A9  (1442) GAATTTGCATGATTCTTCAGCGCTTCTCCTTTGAGCTTTCGC---CTTCC
 DzCYP72A5  (1439) GAATTTGCATGATTCTTCAGTGTTTCTCCTTTGAGCTTTCAC---CTTCC
```

```
DzCYP72A10  (1436) GAATTTGCATGATTATTCAGCACTTCTCATTTGTGCTTTCAC---CTTCC
 DzCYP72A3  (1430) GAATTTGCATGATTATTCAGCACTTCTCATTTGTGCTTTCAC---CTTCC
 DzCYP72A6  (1379) GAATTTGCATGATTATTCAGCACTTCTCATTTGTGCTTTCAC---CTTCC
DzCYP72A11  (1433) GAATCTGCATGATTCTTCAGCACTTTTCCTTTGTGCTTTCGC---TTTCC
 DzCYP72A4  (1436) GAATTTGCATGATTCTTCAGCACTTTTCCTTTGTACTTTCGCTGCTTTCC
 DzCYP72A7  (1340) GAATTTGCATGATTCTTCAGCACTTTTCCTTTGTACTTTCGC---TTTCC
DzCYP72A12  (1436) GAATTTGCATGATTCTTCAGCACTTCTCCTTTGTGCTTTCGC---CTTCC
DzCYP72A13  (1436) GAATTAGCATGATTCTTCAGCACTTCTCCTTCGTGCTCTCGC---CTTCG
                   1501                                              1550
 DzCYP72A1  (1483) TATATCCATGCACCGCACACTGTTATTACTCTTCAACCACAGCATGGGGC
 DzCYP72A8  (1483) TATATCCATGCACCGCACACTGTTATTACTCTTCAACCACAGCATGGGGC
 DzCYP72A2  (1483) --------------------------------------------------
 DzCYP72A9  (1489) TATATCCATGCACCGCACACTGTTATTACTCTTCAACCACAGCATGGGGC
 DzCYP72A5  (1486) TATATCCATGCACCGCACACTGTTATTACTCTTCAACCACAGCATGGGGC
DzCYP72A10  (1483) TATATCCATGCACCGCACTCTGTTATTACTCTTCAACCACAGCATGGAGC
 DzCYP72A3  (1477) TATATCCATGCACCGCACTCTGTTATTACTCTTCAACCACAGCATGGAGC
 DzCYP72A6  (1426) TATATCCATGCACCGCACTCTGTTATTACTCTTCAACCACAGCATGGAGC
DzCYP72A11  (1480) TATATTCATGCACCGCACACTGTTATTACTCTTCAACCACAGCATGGAGC
 DzCYP72A4  (1486) TATATTCATGCACCGCACACTGTTATTACTCTTCAACCAGAGCATGGAGC
 DzCYP72A7  (1387) TATATTCATGCACCGCACACTGTTATTACTCTTCAACCAGAGCATGGAGC
DzCYP72A12  (1483) TATATCCATGCACCGCACAATGTTATTACTCTTCAACCACAGCATGGAGC
DzCYP72A13  (1483) TATATCCATGCACCGCACACTGCTCTTACTCTTCAACCACAGCATGGAGC
                   1551                    1575
 DzCYP72A1  (1533) TCAACTCATGCTGCAAAAGCTGTGA
 DzCYP72A8  (1533) TCAACTCATGCTGCAAAAGCTGTGA
 DzCYP72A2  (1483) -------------------------
 DzCYP72A9  (1539) TCAACTCATGCTGCAAAAGCTGTGA
 DzCYP72A5  (1536) TCAACTCATGCTGCAAAAGCTGTGA
DzCYP72A10  (1533) TCAACTCATGCTGCAAAAGCTGTGA
 DzCYP72A3  (1527) TCAACTCATGCTGCAAAAGCTGTGA
 DzCYP72A6  (1476) TCAACTCATGCTGCAAAAGCTGTGA
DzCYP72A11  (1530) TCAACTCATGCTGCAAAAGTTGTGA
 DzCYP72A4  (1536) TCAACTCATGCTGCAAAAGTTGTGA
 DzCYP72A7  (1437) TCAACTCATGCTGCAAAAGTTGTGA
DzCYP72A12  (1533) TCAACTCATGCTGCAAAAGCTGTGA
DzCYP72A13  (1533) TCAACTCATGCTTCAAAAACTCTGA
```
