# Supplementary material for: The Functional Characterization of DzCYP72A12-4 Related to Diosgenin Biosynthesis and Drought Adaptability in Dioscorea zingiberensis
Source: Int J Mol Sci. 2023 May 8;24(9):8430. doi: 10.3390/ijms24098430 (PMC10179397; doi:10.3390/ijms24098430)
Supplement: Supplementary file 1 [file ijms-24-08430-s001.zip › Table S5.pdf]

Table S5. List of primers used in the present study

Primer names	sequence 5'-3'	Purpose
DzCYP90B71-F	ATGGCGCCGATGGAGCTTCTTCT	Gene clone
DzCYP90B71-R	TCAGGCTGAAGCTTTGTGATCAA	
DzCYP90G6-F	ATGTTTCCTCTAGCTATCATCGT	
DzCYP90G6-R	TCATTCTAGGATGGATAGCTTGC	
DzCYP94N8-F	ATGGAGTTCACTTGGCTTCTCCT	
DzCYP94N8-R	TCAAACAGCTTTGGTACATGGAT	
DzCYP72A12-4-F	ATTTGTTTTGACAGATGGAGTC	
DzCYP72A12-4-R	TCACAGCTTTTGCAGCATGAGTT	
DzCYP90B71-F1	ACTTCTGCAGACCGCCATGGCGCCGATGGA	vector construction
DzCYP90B71-R1	TCACCAATTCAGGCTGAAGCTTTG	
DzCYP90G6-F1	TCTCGAGTCTACCATGTTTCCTCTAGCTATCAT	
DzCYP90G6-R1	AGCTGGTCACCAATTCATTCTAGGATGGATAGCT	
DzCYP94N8-F1	ACAAATCTATCTCTGCCACCATGTGTCTTGCAATGG	
DzCYP94N8-R1	GAGCTGGTCACCAATTCAAACAGCTTTGGTACAT	
DzCYP72A12-4-F1	CAAATCTATCTCTGCCACCATGGAGTCAGTGAT	
DzCYP72A12-4-R1	GAGCTGGTCACCAATTCACAGCTTTTGCAGC	
CAMV35S-F1	AATGGTACCCGACACTCTCGTCTACTCCA	
CAMV35S-R1	GGTAGACTCGAGAGAGATAGATTTGTAG	
UBI1-F1	ACCATTAATTAACCCTGCAGGCTGCAGTGCAGCGTGAC	
UBI1-R1	GGCGGTCTGCAGAAGTAACACCAAACAAC	
NOS-F1	GGTGACCAGCTCGAATTTCCCCGAT	
NOS-R1	CCGATCTAGTAACATAGATGACACCGCGCGC	
CAMV 35s-Pf	GAAGTTCATTTCATTTGGAGAGGAC	positive screen
UBI1-F1	TTGCTTGGTACTGTTTCTTTTGTCGAT	
DzCYP90B71-PR	GCATGCACTTTGATTGGCAAGT	
DzCYP72A12-4-PR	GCATGAGTTGAGCTCCATGCTG	
DzCYP94N8-PR	GTCACAGAGAGCTGAGGCTCGC	
DzCYP90G6-PR	GCTTGCGAACTTTGATAGGAAGGCCCT	
DzGAPDH-qF	TTTGGTGAAAAGTCAGTCGCA	RT-PCR
DzGAPDH-qR	TCGGAGCAGAAATGACAACCT	
DzCYP90B71-qF	GCCGATGGAGCTTCTTCTCAT	
DzCYP90B71-qR	GCCTATCAGAGGCCAGCCCAT	
DzCYP90G6-qF	ATCATCGTCTTGCTATTTCCCA	
DzCYP90G6-qR	CGGCTCGAACAGCTTCCCGTC	
DzCYP94N8-qF	TCCTCCTCCATCTAAACCCAACT	
DzCYP94N8-qR	GACGAGCTCGGTGACGAAGAAG	
DzCYP72A12-4-qF	AAGGAGGCTAAATCCCGGCCTCT	
DzCYP72A12-4-qR	GGTTGATGATCCTTCTGTGTTTGGCCC	
