# Supplementary material for: The Functional Characterization of DzCYP72A12-4 Related to Diosgenin Biosynthesis and Drought Adaptability in Dioscorea zingiberensis
Source: Int J Mol Sci. 2023 May 8;24(9):8430. doi: 10.3390/ijms24098430 (PMC10179397; doi:10.3390/ijms24098430)
Supplement: Supplementary file 1 [file ijms-24-08430-s001.zip › Table S6.pdf]

Table S6. Construction of plant expression vector and specific nucleotide sequences used.

1. Construction of plant expression vector

(1) The pUC57 plasmid was chosen as intermediate clone vector, and was digested with XmaI to produce linear vector. *CAMV35S* promoter, *Ubi1* promoter and *NOS* terminator were amplified by PCR using *pCAMBIA* plasmid and *pBI121* plasmid, respectively. Adjacent fragments were added 15bp overlapping ends by PCR amplification. Gibson Assembly® Master Mix – Assembly (E2611) (NEW ENGLAND Biolabs, Beijing) was used to assemble the targeted nucleotides to produce *pUC57-Pubi1-Tnos* and *pUC57-Pcamv35s-Tnos*.

(2) Sequence of selected genes (*DzCYP97B1*, *DzCYP90G6*, *DzCYP94N8*, *DzCYP72A12-4*) were amplified by PCR and 15 bp overlapping ends were added to both ends of these genes, respectively. Linear vectors of *pUC57-Pubi1-Tnos or pUC57-Pcamv35s-Tnos* were prepared by PCR amplification method too. The same strategy as above was used to produce *pUC57-Pubi1-DzCYP90B71-Tnos* plasmid, *pUC57-Pcamv35s-DzCYP90G6-Tnos* plasmid, *pUC57-Pcamv35s-DzCYP90N8-Tnos* plasmid and *pUC57-Pcamv35s-DzCYP72A12-4-Tnos* plasmid.

(3) Linear pCAMBIA plasmid was produced by SbfI digestion and nucleotide purification. Pubi1-DzCYP90B71-Tnos fragment was amplified with 15 bp overlapping ends added. Gibson assembly was used to produce *pCAMBIA-Pubi1-DzCYP90B71-Tnos*, named *pCAMBIA-DzCYP90B71*. In the meantime, two restriction endonuclease recognition sites (SbfI & NruI) were added flanking the cassette facilitating multi-cassettes assembly. The *pCAMBIA-DzCYP72A12-4* construct was created in the same way.

(4) Linear *pCAMBIA-DzCYP90B71* was produced by digestion of NruI, and assembled with Pcamv35s-DzCYP90G6-Tnos cassette using Gibson Assembly method the same as above to produce *pCAMBIA-Pubi1-DzCYP90B71-Tnos-Pcamv35s-DzCYP90G6-Tnos*, named *pCAMBIA - DzCYP90B71/DzCYP90G6*.

(5) Linear *pCAMBIA -DzCYP90B71/DzCYP90G6* was created by digestion of PmeI, and assembled respectively with *Pcamv35s-DzCYP90N8-Tnos* cassette and *Pcamv35s-DzCYP72A12-4-Tnos* cassette to produce *pCAMBIA-DzCYP90B71/DzCYP90G6/DzCYP90N8* and *pCAMBIA-DzCYP90B71/DzCYP90G6/DzCYP72A12-4*.

2. Sequences used in expression vector construction.

>CAMV 35S promoter

Cgacactctcgtctactccaagaatatcaaagatacagtctcagaagaccaaagggctattgagacttttcaacaaagggtaatatcgggaaa
cctcctcggattccattgcccagctatctgtcacttcatcaaaaggacagtagaaaaggaaggtggcacctacaaatgccatcattgcgataaa
ggaaaggctatcgttcaagatgcctctgccgacagtggtcccaaagatggacccccacccacgaggagcatcgtggaaaaagaagacgttc
caaccacgtcttcaaagcaagtggattgatgtgataacatggtggagcacgacactctcgtctactccaagaatatcaaagatacagtctcaga
agaccaaagggctattgagacttttcaacaaagggtaatatcgggaaacctcctcggattccattgcccagctatctgtcacttcatcaaaagga
cagtagaaaaggaaggtggcacctacaaatgccatcattgcgataaaggaaaggctatcgttcaagatgcctctgccgacagtggtcccaaa
gatggacccccacccacgaggagcatcgtggaaaaagaagacgttccaaccacgtcttcaaagcaagtggattgatgtgatatctccactga
cgtaagggatgacgcacaatcccactatccttcgcaagacccttcctctatataaggaagttcatttcatttggagaggacacgctgaaatcacca
gtctctctctacaaatctatctct

>Ubi1 promoter (*Zea Mays*)

ctgcagtgcagcgtgacccggtcgtgcccctctctagagataatgagcattgcatgtctaagttataaaaaattaccacatattttttttgtcacact
tgtttgaagtgcagtttatctatctttatacatatatttaaactttactctacgaataatataatctatagtactacaataatatcagtgttttagagaatca

tataaatgaacagttagacatggtctaaaggacaattgagtattttgacaacaggactctacagttttatctttttagtgtgcatgtgttctcctttttttt
tgcaaatagcttcacctatataatacttcatccattttattagtacatccatttagggtttagggttaatggtttttatagactaattttttttagtacatctatt
ttattctattttagcctctaaattaagaaaactaaaactctattttagttttttttatttaataatttagatataaaatagaataaaataaagtgactaaaaatt
aaacaaataccctttaagaaattaaaaaaactaaggaaacatttttcttgtttcgagtagataatgccagcctgttaaacgccgtcgacgagtcta
acggacaccaaccagcgaaccagcagcgtcgcgtcgggccaagcgaagcagacggcacggcatctctgtcgctgcctctggacccctctc
gagagttccgctccaccgttggacttgctccgctgtcggcatccagaaattgcgtggcggagcggcagacgtgagccggcacggcaggcg
gcctcctcctcctctcacggcacggcagctacgggggattcctttcccaccgctccttcgctttcccttcctcgcccgccgtaataaatagacac
cccctccacaccctctttccccaacctcgtgttgttcggagcgcacacacacacaaccagatctcccccaaatccacccgtcggcacctccgc
ttcaaggtacgccgctcgtcctccccccccccccctctctaccttctctagatcggcgttccggtccatggttagggcccggtagttctacttctgt
tcatgtttgtgttagatccgtgtttgtgttagatccgtgctgctagcgttcgtacacggatgcgacctgtacgtcagacacgttctgattgctaactt
gccagtgtttctctttggggaatcctgggatggctctagccgttccgcagacgggatcgatttcatgattttttttgtttcgttgcatagggtttggttt
gcccttttcctttatttcaatatatgccgtgcacttgtttgtcgggtcatcttttcatgcttttttttgtcttggttgtgatgatgtggtctggttgggcggtc
gttctagatcggagtagaattctgtttcaaactacctggtggatttattaattttggatctgtatgtgtgtgccatacatattcatagttacgaattgaa
gatgatggatggaaatatcgatctaggataggtatacatgttgatgcgggttttactgatgcatatacagagatgctttttgttcgcttggttgtgat
gatgtggtgtggttgggcggtcgttcattcgttctagatcggagtagaatactgtttcaaactacctggtgtatttattaattttggaactgtatgtgtg
tgtcatacatcttcatagttacgagtttaagatggatggaaatatcgatctaggataggtatacatgttgatgtgggttttactgatgcatatacatga
tggcatatgcagcatctattcatatgctctaaccttgagtacctatctattataataaacaagtatgttttataattattttgatcttgatatacttggatga
tggcatatgcagcagctatatgtggatttttttagccctgccttcatacgctatttatttgcttggtactgtttcttttgtcgatgctcaccctgttgtttgg
tgttacttctgcag

>NOS terminater

Gatcgttcaaacatttggcaataaagtttcttaagattgaatcctgttgccggtcttgcgatgattatcatataatttctgttgaattacgttaagcatg
taataattaacatgtaatgcatgacgttatttatgagatgggtttttatgattagagtcccgcaattatacatttaatacgcgatagaaaacaaaatat
agcgcgcaaactaggataaattatcgcgcgcggtgtcatctatgttactagatc

>DzCYP90B71

atggcgccgatggagcttcttctcatcgtctctccactagtgctagccctcatcatcttcttcagcttcagagggaccagcaagggcggcgaca
aggcggaaaagatccctcccggcacgatgggctggcctctgataggccacacgatccccttcatgcagccccactcctccgcctctctcggc
ctcttcgtcgaccaaaatatcgccaagcatgggaggattttcaggatgaacttgttgggaaagccaacgatcgtctccgcagacgctgatttca
atcggttcattcttcagagcgagggccgaatgttcgagaacagctgcccgacgagtatcgcagagatcatggggcggtggtcgatgctagcg
ctcgctggtgacgtgcaccgcgagatgcggtccatcgcggtgaacttcatgagcaatgtcaagctccggacctacttccttcccgacgtcgag
cagcaggccatcaagatcctcagcgcctggcgccatggctccaccttctctgcacaagaggagggcaagaagttcgccttcaatctgatggt
gaagcatctgatgagcatggacccgggaatgccggagacggagcaactgaggaaggagtacattaccttcatgaagggcatggcctccatc
cctttgaacttgccaggaactgcttataggaaagccttgcagtcaagatccataatcctgaaaataatggggcaaaagctggatgagagggtg
gagaaggtcaagaggggatgtgaaggccttgaagaagatgaccttcttgcatcagttgctgctcaatcaaacatcacaagagatcaaattctc
gacctgatactcagcatgctctttgctggccatgagacgtcctctgccgccatttgcctcgccatctacttccttgagtcttctcccaaagctcttca
acaacttcgagaggagcacatcaacatagccaaaatgaagaaggagaaaggagagactgggcttacatgggatgactacaaacagatgga
gttcactcactgtgtgatcaatgaaactcttaggcttggcaacattgtgaagttcttgcacaggaaggccattaaggatgtccaatacaaaggtta
tgatattccttgtggatgggaagttgtccctataatctcctccgcacatttggatccctccatttatgacgatccacagtcctacaatccttggagat
ggcagacaatctcaacagcgacatcaaagaacaacaatatcatgtcattcagcggcggtcctcgtctgtgccccggggccgagctcgcaaa
gatggagatggctgtcttcctgcaccaccttgtccagaagttcaactgggagttggctgagcatgactaccctgtatccttcccatttctagggtt
ccccaagcacttgccaatcaaagtgcatgcaattgatcacaaagcttcagcctga

>DzCYP90G6

atgtttcctctagctatcatcgtcttgctatttcccacactgctgctcctcttcataggagtggccctgggtttgagaagtggagccaatgagagct
ggaagaagagggggctcaacatccctccaggaagcatgggctggccgctcctcggcgagaccatcgccttccggaagctccatccctgca
cctctctcggcgagtacatggaggatcgtctccagaggtatggaaagatctaccgctcgaacttgttcggcgcgccgacggtggtttcggcgg

atgcagagctgaaccggttcgtgctgatgaacgacgggaagctgttcgagccgagctggccgaagagcgtggcggacatactgggaaaga
cgtcgatgctggtgctcacaggggagatgcatcgctacatgaagtccttgtccgtcaacttcatggggatcgctaggcttcggaatcacttcctt
ggggactctgagcgctatatcttggagaaccttgcgacctggaaggagggcgttcctttccctgctaaagaagaagcttgcaagataaccttca
atttaatggtgaagaacatactgagtatgaatcctggggagccagagaccgagaggttgcgcattctctacatgtccttcatgaagggagtgat
agctatgcctctcaatttccctggaactgcatacaggaaagccattcagtctagagctacaatcctgaaaaccattgagcatttgatggaggata
ggctggagaagaagaaggcaggcactgataatatcggagaagctgatcttctaggtttcattctagagcagtcgaacttggatgctgaacaatt
cggagacttgctgttaggtttgctttttggtggccatgagacctcctccactgccatcactctggctatctacttccttgaaggatgccctaaagct
gtacaagaactaagggaagagcatttgaacctggtgaggatgaagaagcagagaggagagtccaaagcactcacttgggaagactacaaa
tccatggactttgcacagtgtgtggtgagtgagactctaaggctgggaaacatcatcaagtttgtgcacaggaaggctaacactgatgtccaatt
taaaggatatgacataccgagtggctggagtgtgattccggtgttcgccgcagctcatttagatcctactgtctatgacaatcctcagaaatttgat
ccttggagatggcagacaatctcctccagcactgctaggattgacaattacatgccattcggtcaggggctgcgcaactgtgctggccttgagc
tcgccaagatggagatcgccgtgttccttcaccaccttgtccttaacttcgactgggagcttgctgagccagatcaccccctcgcctacgccttc
cctgaattcgaaaagggccttcctatcaaagttcgcaagctatccatcctagaatga
>DzCYP94N8
atggagttcacttggcttctcctcctcgtcctcttcatcaccaccaccatcttcctcctcctccatctaaacccaactcccacccccaacttcacccc
tctaaaaccctacccaatcctccaaaacctcccccacttagtcaaaaactcccaccgtctcctcttcttcgtcaccgagctcgtctcctcctctccc
tcctccacctccaccctcatccccttcgtcttcacctccaacccctccaacgtcgaacacatgctccgctccaacttccccaactacatcaaagg
ctcttccgtcatctccactctgcacgacttcctcggcgacggcatcttcaactccaacggtcctctctggcgcctccagcgcaagaccgccagc
ttcgagttcaacaccaagtccctccgctccttcatcttccaccacgtccgccacgaatccctccacgctctcctccccattttatcgaacacctcc
cgtgcatccctccccgtcctcatcgacctccaggacctcctcgaacgcttcgccttcgacaacgtctgctccctcgtcttcggccacgaccccc
gctgcctccacgactccgccgacggcctccgcttcttccacgccttccaggaggcctcccatctcagcatcgagcggatgaaccacgcctttg
atcttctttggaaggtcaacaagtggctcaacgtcggctctgagcgccgtttaaaccattcgttgttgatcgttcgtgagtacgcttcgagattcgt
gtccttgcggaagacccaagccggtgacgacctcctctcccgtttcgccgcggatgagaccatctccgatgacctccttgtcgacatcctcattt
gcttcgtcctcgccggccgggacaccacccccgccgcgctctcctggttcttctggctcctctcgtcccgcccggacgtttcgcggaacatctt
ggtcgagattcagtctatccgagctcgatccggcgaccgtgatggcgatcggttcttcagtctggaggagctgagagagatgaactacctcca
cgccgcgctatccgaggcgatgagactctaccctccggtgccactcctgccgagatgcgccgccgaggacgacgtcctcccggatgggac
cgtcgtgaagaaaggctggaccttgatgtacaacgcctacgccatggggaggatggagagcatttggggcaaggactgcatggagatgag
gccggagaggtggctggaggacggcgtcttccagccgacgagcccgttccggtacccggtgttcctcggcgggccgaggatgtgcttggg
caaggagatggcttacatccagatgaaggcggtggcggcctgcattctcgagaagttcgacatcgacgtcgtcggcgcctccggcgagcct
cagctctctgtgacgatgacgatgaagggtggcttgccagtgaggatcaaagagagaaatccatgtaccaaagctgtttga
>DzCYP72A12-4
atggagtcagtgatgggagtgatatgggcggtggcggcggtggtggtagtggcggcggcgtggaggacattggattggatttggtggacac
cgaggaggctggaccgggagctccggcggcagggcctgcgcggcaaccagtaccgagtcttgcacggcgatctcaaggaaaacgtccg
gctctcgaaggaggctaaatcccggcctctgcctcttcactgccatgacatcgccccccgcgttctccctctcttccacaacgccatcaaagat
cacggtgaaaagtgggccaaacacagaaggatcatcaaccctgcattccatcttgagaaactaaagctaatgctgccagcattctctacatctt
gtggtgaactgattagaagatgggagaagatgattcctaatgaaggctcccaagaactaaatgtctttccagagctccaagacctcacaaaag
atgtcatctccaggactgcattcggtagcagttatgaagacggaagaagaatatttgaactcctaacagagcaaattcagcttcttattccagcttt
ccagactgtatacattcctggttatcgatttctgcccacaccaatgaacaaaagaagcagccaagtgtacaatgagatgaaaagaattcttatag
gcatgattgagaagagagaaaaggccataagaatgggggaaagtagcaagaatgaccttctgggtttgttactagactccaatatcaaagag
agtgaagtgcatgggaagtcccaaaacaaagggatgagcactgaggatgtggttgaagagtgcaagctgttctactttgcagggcaagaga
ctacatcacttctactcacatggacaatgattttattgagcatgtatccaaattggcaggctaaggcaagagaagaggttcttcaagtctttggaaa
gagcgcaccagatatggagggattgagccacttgaagattgtgaccatgattctatatgaagttctaaggttatatccaccagcggttttcctaac
aagaaaaacctacaaagcaatggaactcggtggaattacttaccctccaggagtgatactctcactgcctctactcttcattcaccatgaccctgt
tttctggggagaagacgccaaagagtttaatccagagaggttttccgaagggatatcgaaagcatccaaagttccgggtgccttctttcctttcg

gtggaggtccgcgcatttgcattggccaaaactttgctctgattgaagctaagataggaatttgcatgattcttcagcacttctcctttgtgctttcg
ccttcctatatccatgcaccgcacaatgttattactcttcaaccacagcatggagctcaactcatgctgcaaaagctgtga

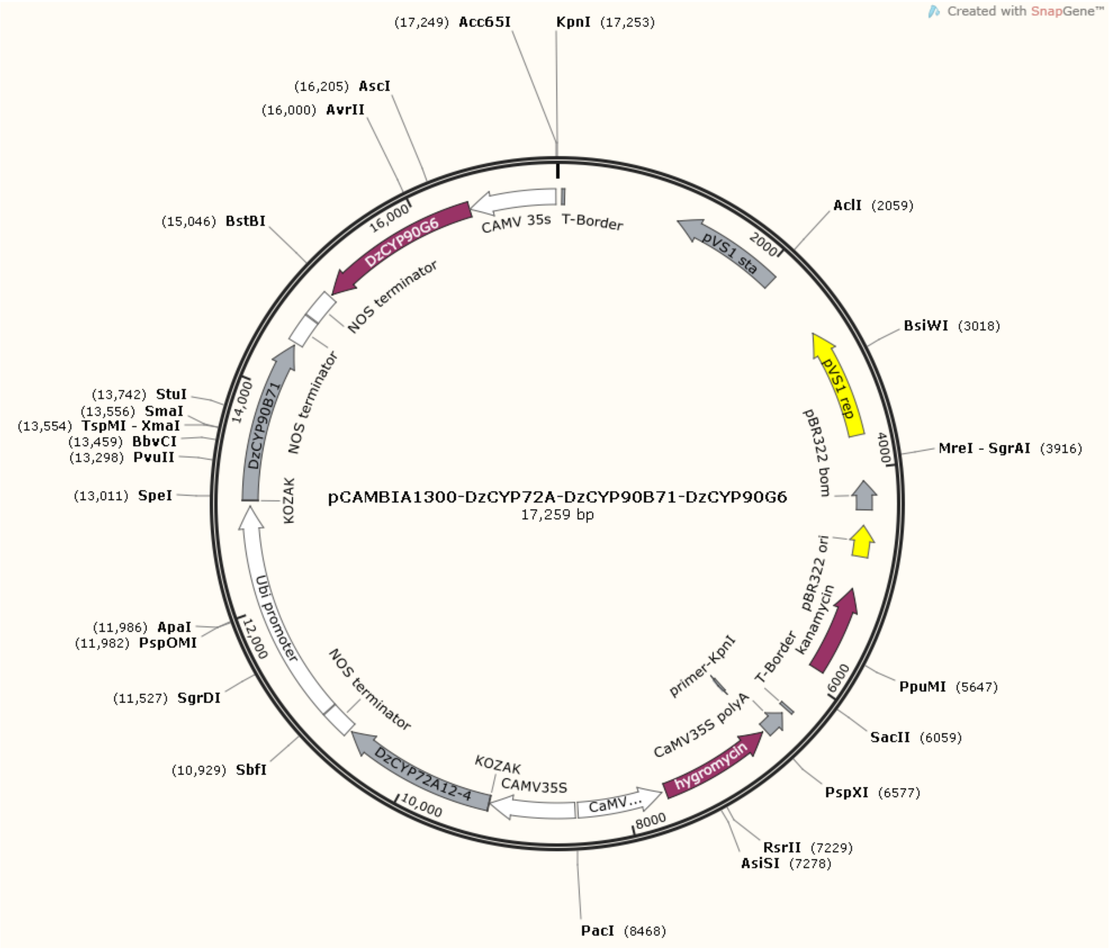

Diagram of pCAMBIA-DzCYP72A12-4/DzCYP90B71/90G6

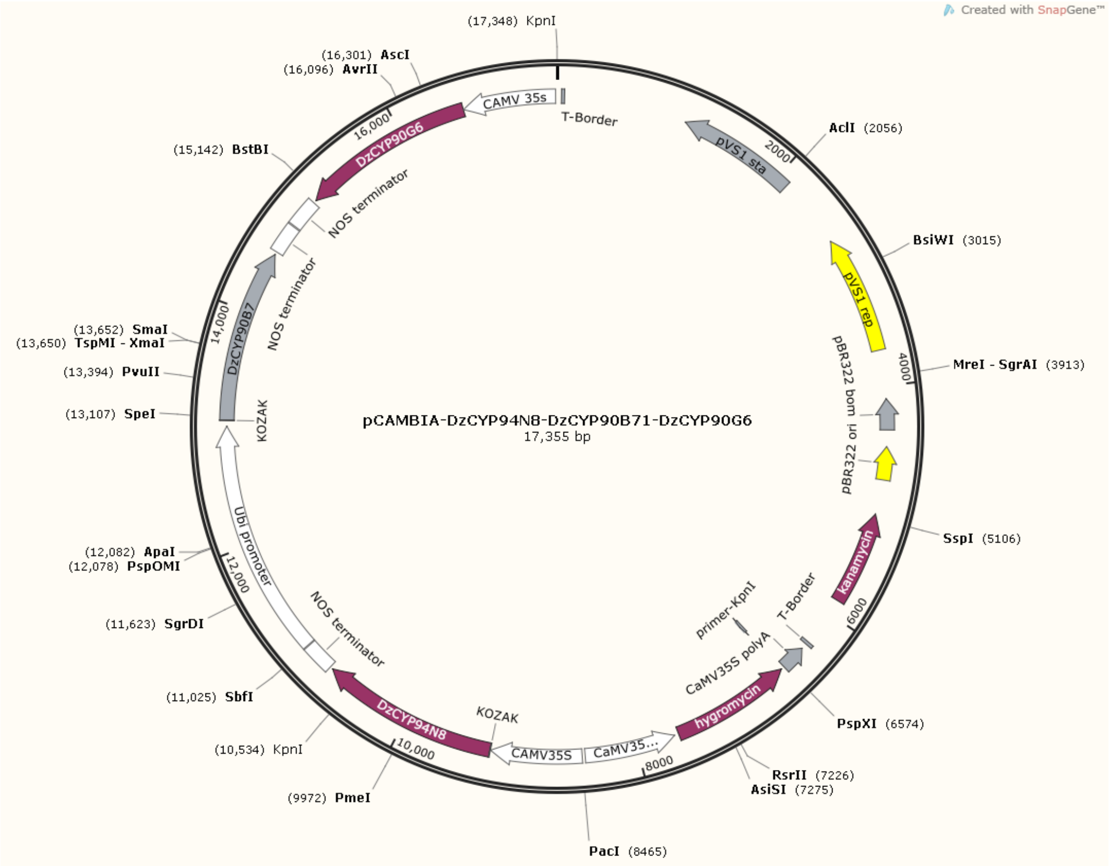

Diagram of pCAMBIA-DzCYP94N8/DzCYP90B71/90G6

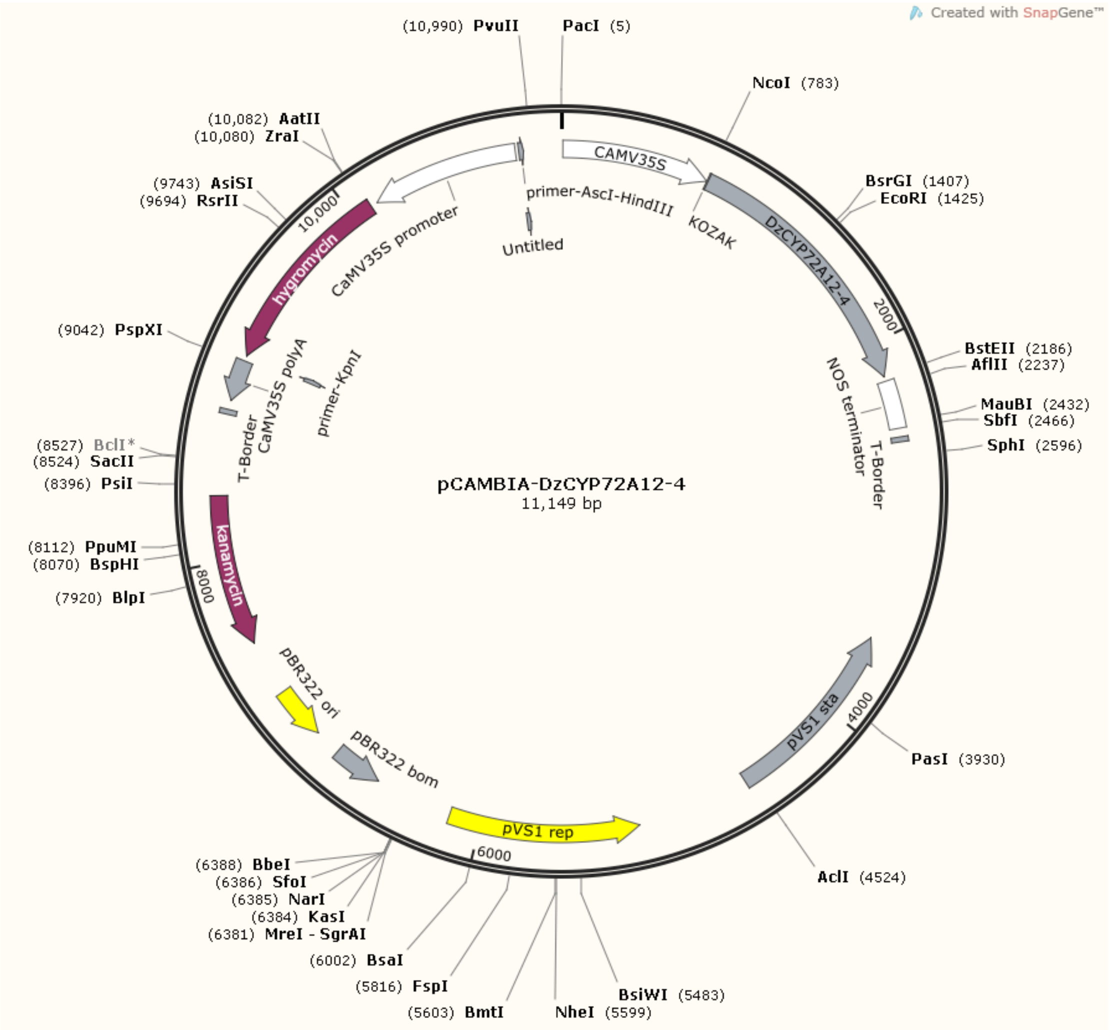

Diagram of pCAMBIA-DzCYP72A12-4

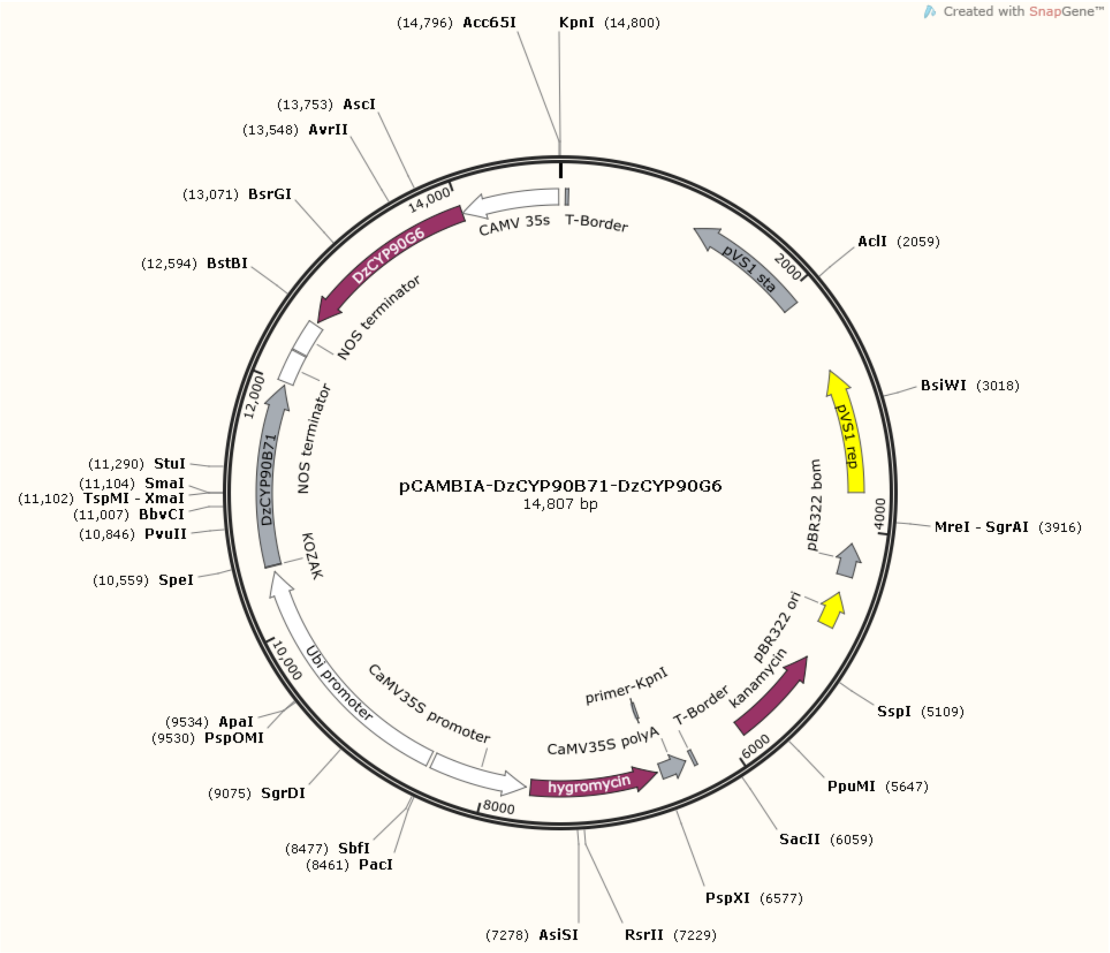

Diagram of pCAMBIA- DzCYP90B71/90G6
